# Integrative Taxonomic Revision of Goatfishes (Mullidae) in the Northern South China Sea Based on Morphology and Multilocus Molecular Data

**DOI:** 10.1002/ece3.73378

**Published:** 2026-04-10

**Authors:** Xiafang Li, Zhisen Luo, Murong Yi, Bin Kang, Xiongbo He, Liangliang Huang, Hung‐Du Lin, Yunrong Yan

**Affiliations:** ^1^ College of Fisheries Guangdong Ocean University Zhanjiang China; ^2^ Fisheries College Ocean University of China Qingdao China; ^3^ Guangdong Provincial Engineering and Technology Research Center of Far Sea Fisheries Management and Fishing of South China Sea Guangdong Ocean University Zhanjiang China; ^4^ College of Environmental Science and Engineering Guilin University of Technology Guilin China

**Keywords:** integrative taxonomy, morphological diagnosis, Mullidae, northern South China Sea, species delimitation

## Abstract

The goatfishes (Mullidae) are widely distributed in tropical and subtropical seas, yet taxonomic resolution in the northern South China Sea remains limited. We conducted an integrative taxonomic revision combining morphological characteristics with multilocus molecular markers (mitochondrial COI, 16S rRNA, and nuclear IRBP) for 283 mullid specimens collected from the northern South China Sea, providing updated species‐level diagnoses and a dichotomous identification key for the regional fauna. Morphological examination identified 18 species belonging to three genera, and principal component analysis (PCA) revealed intergeneric differences in quantitative traits, with depth‐related and head‐region variables contributing strongly to genus‐level separation despite partial overlap in morphospace. Molecular phylogenetic analyses showed that some single‐locus trees provided limited resolution at certain nodes, whereas the concatenated COI + 16S rRNA dataset recovered monophyly for all examined species with higher support. Species delimitation results indicated that Automatic Barcode Gap Discovery (ABGD) and Bayesian Poisson Tree Processes (bPTP) were concordant, recovering 22 molecular operational taxonomic units (MOTUs) across the full COI dataset, including four MOTUs represented only by GenBank reference sequences; among our sampled specimens, the 18 morphologically identified species each corresponded to a single MOTU. Based on integrated morphological and multilocus evidence, we revised the diagnoses and diagnostic characters for three genera and 18 goatfish species from the northern South China Sea and compiled a dichotomous identification key, thereby clarifying species boundaries within Mullidae, improving identification accuracy, and supporting future surveys, biodiversity inventories, and monitoring in this region.

## Graphical abstract

We performed an integrative taxonomic revision of Mullidae from the northern South China Sea using morphology and multilocus markers (COI, 16S rRNA, and IRBP) based on 283 specimens. Morphology identified 18 species in three genera, with PCA showing intergeneric differentiation mainly driven by depth‐related and head‐related traits despite partial overlap. Molecular analyses provided stronger species‐level support with concatenated COI + 16S rRNA, and ABGD and bPTP were concordant, recovering 22 COI‐based MOTUs (including four represented only by GenBank), while all 18 sampled morphological species each corresponded to a single MOTU.

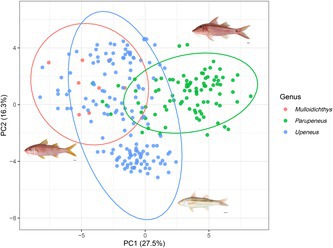

## 1 Introduction

Accurate species identification and taxonomy provide the foundation for biodiversity assessments, ecological research, and fisheries management (Baerwald et al. 2020; Fischer 2013; Livingstone 2016; Walton et al. 2025). Misidentifications, unresolved synonymy, and inconsistent records in historical literature may be propagated through subsequent studies and data syntheses, ultimately undermining estimates of species composition and the management decisions based on them (Bortolus 2008). These problems are particularly acute in taxonomic groups undergoing active revision and ongoing species descriptions, such as goatfishes (Mullidae). Mullidae are widely distributed in tropical and subtropical seas and currently comprise six genera and 105 recognized species worldwide (Uiblein et al. 2024, 2025). The family is readily diagnosed by a pair of hyoid barbels located on the lower jaw, which constitute a primary diagnostic feature in identification and systematics (Kim et al. 2001; Uiblein 2007). In recent years, ongoing descriptions of new taxa and taxonomic revisions have further refined species identification and classification within Mullidae (Fernandez‐Silva et al. 2016; Uiblein et al. 2017, 2020, 2025; Uiblein and Motomura 2021). However, integrative taxonomic research and species identification of goatfishes in Chinese waters remain limited, and historical sources contain multiple taxonomic errors and inconsistent records. Consequently, these issues can compromise regional biodiversity assessments and management decisions, underscoring the need for a systematic revision based on updated morphological and molecular evidence.

Along the Chinese coast, goatfishes are reported primarily from the northern South China Sea (NSCS), whereas only a few species have been reported from the East China Sea and the Yellow Sea. Taxonomic research on Mullidae in China has largely relied on historical literature, with few comprehensive revisions or evidence‐based updates in recent decades, resulting in ongoing controversies regarding species identification and distribution records. This situation has been further aggravated by delayed taxonomic updates and the slow incorporation of newly described species and newly documented records. First, historical misidentifications are evident. Chinese records of *Parupeneus trifasciatus* actually represent records misidentified as *P. multifasciatus* (Institute of Zoology, Chinese Academy of Sciences et al. 1962; Cheng and Zheng 1987; Shen 1993). Randall and Myers (2002) reported that *P. trifasciatus* is restricted to the Indian Ocean; therefore, previous Chinese records of this species require re‐evaluation. Chinese records of *Upeneus subvittatus* (Temminck & Schlegel, 1843) describe a lateral body stripe (Institute of Zoology, Chinese Academy of Sciences et al. 1962); however, recent taxonomic accounts and a recent record describe the body as lacking lateral stripes (Choi et al. 2024; Uiblein and Causse 2013). Therefore, the occurrence of *Upeneus subvittatus* in Chinese coastal waters requires further verification. Second, confusion between closely related species has been frequent. *Parupeneus ciliatus* and *P. biaculeatus* are highly similar morphologically (Tashiro and Motomura 2015) and have often been misidentified in coastal records from China (Institute of Zoology 1962; Liu et al. 2016), resulting in inconsistent distribution information. *Upeneus sundaicus* has also often been misidentified as *Mulloidichthys vanicolensis* (Chen and Zhang 2015; Yan et al. 2021). Third, questionable records and unresolved taxonomic status remain. The original description of *U. luzonius* Jordan & Seale, 1907, as recorded in China, is ambiguous, and Uiblein et al. (2019) considered it to represent a distinct species, *U. heterospinus*. Records of *P. jansenii* , *P. forsskali* , and *M. pfluegeri* in China are also disputed (Wu and Zhong 2021; Liu et al. 2024), and should be re‐evaluated using combined morphological and molecular evidence. Collectively, these historical misidentifications, confusion among closely related species, and questionable records indicate that the species composition of Mullidae along the Chinese coast, particularly in the northern South China Sea, remains unclear, and that a systematic reassessment and revision based on morphological and molecular data are urgently warranted.

Traditional morphology has provided the taxonomic foundation for Mullidae, with species diagnoses commonly based on a combination of meristic and morphometric traits, particularly gill‐raker counts, coloration patterns, barbel length, fin morphology and banding, and body proportions such as caudal‐peduncle depth, interorbital width, and interdorsal distance (Randall and Myers 2002; Uiblein and Heemstra 2010; Uiblein and Causse 2013; Uiblein et al. 2025). Despite their utility, some diagnostic characters show ontogenetic variation, and coloration may differ among geographic populations, complicating morphology‐based identification. In the Gulf of Thailand, black and red morphs of *U. tragula* have been linked to stages of sexual maturity (Pavlov and Emel'yanova 2018), whereas *U. guttatus* shows geographic variation in barbel coloration across its range (Uiblein and Heemstra 2011). Such ontogenetic and geographic variation can increase the risk of misidentification; consequently, molecular evidence has been increasingly incorporated into Mullidae taxonomy. Since the proposal of DNA barcoding by Hebert et al. (2003), mitochondrial markers such as COI have been widely applied to fish identification and the construction of barcode reference libraries (Antil et al. 2023). However, morphological divergence is not always concordant with mitochondrial divergence: Keskin and Can (2009) showed that although *Mullus barbatus ponticus* can be recognized morphologically as a subspecies of *Mullus barbatus* , mitochondrial loci (Cyt b, 12S rRNA, and COII) did not provide sufficient differentiation to support subspecies status. Accordingly, integrative studies in Mullidae have combined COI with additional markers and morphology to validate closely related taxa and to flag potential cryptic lineages (Ahmed et al. 2016; Echreshavi et al. 2023; Uiblein and Gouws 2014, 2015). Beyond mitochondrial markers, nuclear genes provide complementary evidence because mitochondrial DNA is maternally inherited and largely nonrecombining, whereas nuclear loci are biparentally inherited and recombining (Betancur‐R et al. 2013). Comparing these marker types can therefore help identify mitonuclear discordance, including patterns potentially associated with introgression or hybridization, and reduce overreliance on a single maternal genealogy (Toews and Brelsford 2012). The nuclear gene Interphotoreceptor Retinoid‐Binding Protein (IRBP), in particular, has been used in teleost phylogenetics and can also be informative at relatively shallow taxonomic scales for evaluating species relationships and testing monophyly (Dettaï and Lecointre 2008; Chen and Mayden 2012). Therefore, in addition to the mitochondrial genes COI and 16S rRNA, we included IRBP as a nuclear marker to complement mitochondrial evidence and to assist in assessing species monophyly. Finally, given the limited resolution of single‐gene datasets at deeper nodes and the potential effects of heterogeneous sequence quality in public databases on identification accuracy (Wong et al. 2011), we adopted a multilocus approach to strengthen species identification and COI‐based species delimitation inferences.

Species delimitation is often approached within an integrative taxonomy framework, which emphasizes combining multiple lines of evidence rather than relying on a single criterion (Padial et al. 2010; Fujita 2012; Maddison and Whitton 2023). In Mullidae, phylogenomic analyses combined with quantitative morphology have provided a robust framework for clarifying interspecific relationships and assessing how body form varies across lineages and genera (Nash et al. 2022), while morphometric studies integrating ecological proxies have further documented ecomorphological differentiation among co‐occurring goatfish species (Mittelheiser et al. 2022, 2025). Nevertheless, despite these advances, region‐specific integrative revisions remain scarce for the NSCS, where most Chinese records of goatfishes originate, and where several taxa still lack clearly delimited species boundaries and stable diagnostic characters. Accordingly, a focused reassessment based on systematic voucher‐backed sampling and multilocus validation is needed to resolve the composition of goatfishes in this region.

Based on systematic sampling of Mullidae from the northern South China Sea, we conducted a taxonomic revision by integrating morphological evidence with multilocus markers, including mitochondrial COI and 16S rRNA genes, and the nuclear gene IRBP. The objectives of this study are to (1) reassess the regional species composition using diagnostic morphological characters, provide updated species‐level diagnoses, and develop an identification key for the NSCS fauna; (2) quantify and compare intergeneric body‐shape differences using morphometric data to characterize genus‐level morphological differentiation; and (3) reconstruct phylogenetic relationships using mitochondrial (COI, 16S rRNA) and nuclear (IRBP) loci, test species monophyly, and conduct COI‐based species delimitation to evaluate concordance between molecular partitions and morphology‐based species hypotheses. By correcting misidentifications and questionable records in the literature, this revision clarifies species diversity and taxonomic boundaries of Mullidae in the region and provides an identification key to support biodiversity assessment, resource monitoring, and conservation management.

## 2 Materials and Methods

### 2.1 Sample Collection and Identification

**FIGURE 1:**
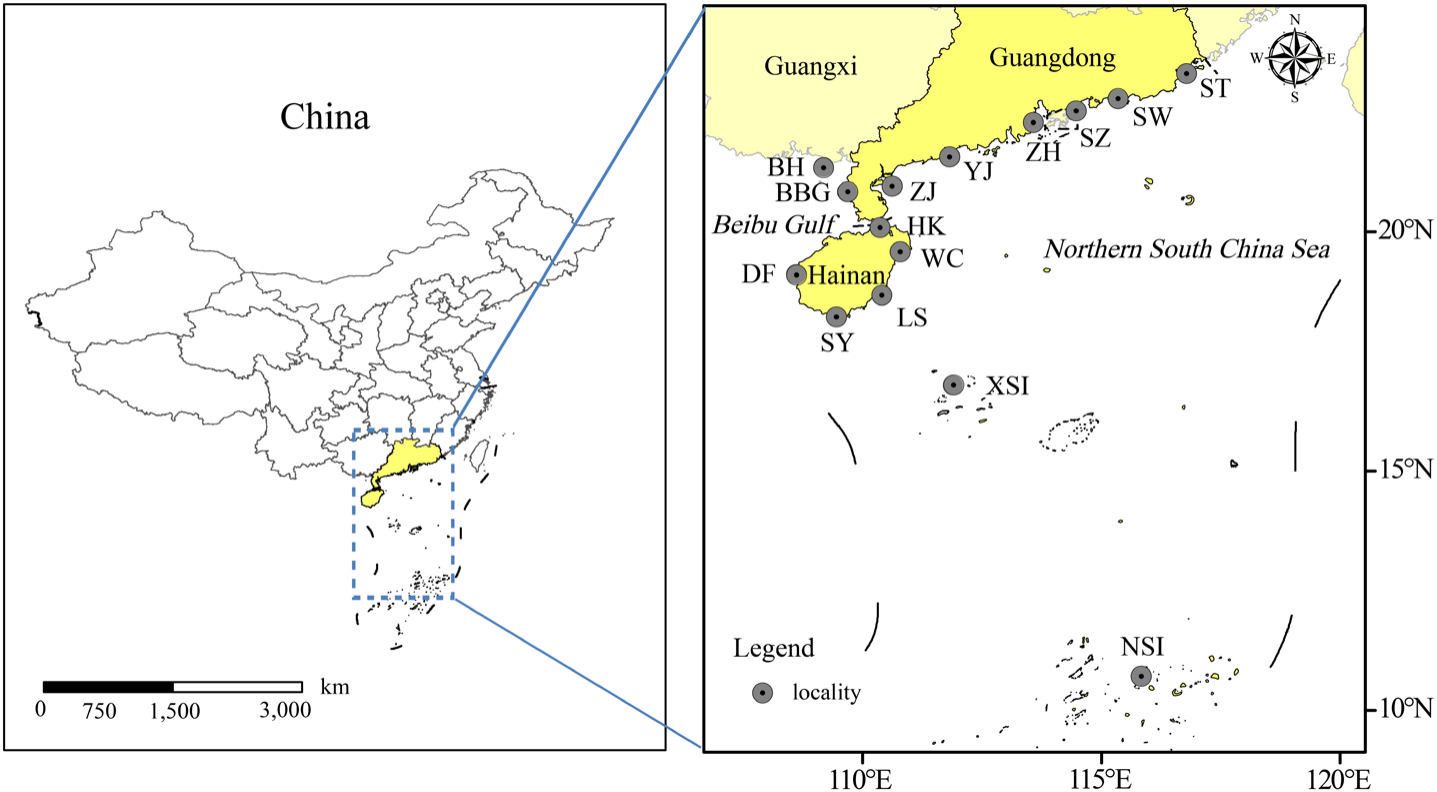
Sampling localities in the northern South China Sea (NSCS), China. Sampling localities were concentrated along the Guangdong, Guangxi, and Hainan coasts of the northern South China Sea, including the Beibu Gulf, the Xisha Islands, and the Nansha Islands. Abbreviations denote sampling localities: ST (Shantou, Guangdong, China), SW (Shanwei, Guangdong, China), SZ (Shenzhen, Guangdong, China), ZH (Zhuhai, Guangdong, China), YJ (Yangjiang, Guangdong, China), ZJ (Zhanjiang, Guangdong, China), HK (Haikou, Hainan China), WC (Wenchang, Hainan, China), LS (Lingshui, Hainan, China), SY (Sanya, Hainan, China), DF (Dongfang, Hainan, China), BBG (the Beibu Gulf, China), BH (Beihai, Guangxi, China), XSI (the Xisha Islands, China), NSI (the Nansha Islands, China).

A total of 283 goatfish specimens were collected from the NSCS between March 2014 and October 2021 (Figure 1). Sampling localities were concentrated along the Guangdong, Guangxi, and Hainan coasts of the northern South China Sea, including the Beibu Gulf, the Xisha Islands, and the Nansha Islands. Collection methods included gill netting, spearfishing, cast netting, and angling. After collection, all samples were frozen as soon as possible for subsequent morphological examination and molecular analysis (Table 1). Voucher specimens were deposited at the College of Fisheries, Guangdong Ocean University (Zhanjiang, China).

**TABLE 1:** Sampling summary and dataset composition.

Genus	Species	Examined and measured		Morphometrics used in multivariate analyses		Number sequenced		
		Number	SL range (mm)	Number	SL range (mm)	COI	16S rRNA	IRBP
*Mulloidichthys*	*M. flavolineatus*	5	138.6–213.7 (174.3 ± 27.7)	4	138.6–213.7 (174.1 ± 32.0)	5	5	3
	*M. vanicolensis*	6	145.6–250.3 (218.2 ± 38.3)	4	145.6–250.3 (213.5 ± 47.1)	5	5	3
*Parupeneus*	*P. barberinoides*	2	163.1–177.3 (170.2 ± 10.0)	2	163.1–177.3 (170.2 ± 10.0)	1	1	1
	*P. barberinus*	5	268.2–343.3 (303.4 ± 32.4)	2	271.9–343.3 (307.6 ± 50.5)	5	5	5
	*P. biaculeatus*	32	114.5–217.0 (150.4 ± 19.5)	26	123.8–217.0 (153.7 ± 18.9)	5	5	3
	*P. chrysopleuron*	24	113.3–177.0 (134.7 ± 13.0)	24	113.3–177.0 (134.7 ± 13.0)	5	5	5
	*P. ciliatus*	4	132.0–203.4 (178.9 ± 32.0)	2	132.0–187.7 (159.9 ± 39.4)	3	3	2
	*P. crassilabris*	2	236.4–250.4 (243.4 ± 9.9)	2	236.4–250.4 (243.4 ± 9.9)	2	2	2
	*P. cyclostomus*	4	206.6–330.2 (290.1 ± 57.4)	2	298.3–330.2 (314.3 ± 22.6)	5	5	3
	*P. heptacantha*	5	129.6–182.0 (161.8 ± 20.9)	2	176.0–182.0 (179.0 ± 4.2)	5	4	2
	*P. indicus*	16	111.1–247.8 (188.1 ± 43.7)	14	120.6–247.8 (192.5 ± 41.4)	5	5	5
	*P. multifasciatus*	14	119.5–189.1 (152.7 ± 24.4)	14	119.5–189.1 (152.7 ± 24.4)	5	5	3
*Upeneus*	*U. heterospinus*	18	94.0–130.5 (112.5 ± 10.6)	12	94.0–130.5 (113.2 ± 12.3)	8	7	3
	*U. itoui*	5	101.1–105.2 (103.2 ± 1.7)	3	102.9–105.2 (104.2 ± 1.2)	4	4	4
	*U. japonicus*	39	89.1–142.9 (104.0 ± 10.8)	37	89.1–142.9 (103.9 ± 11.0)	5	5	4
	*U. sulphureus*	63	95.0–150.5 (124.0 ± 14.2)	63	95.0–150.5 (124.0 ± 14.2)	6	6	2
	*U. sundaicus*	8	101.5–146.5 (120.3 ± 16.7)	6	101.5–124.5 (112.8 ± 10.7)	6	6	6
	*U. tragula*	31	88.5–198.5 (133.7 ± 30.7)	30	88.5–198.5 (134.2 ± 31.2)	5	5	2
Total		283	/	249	/	85	83	58

*Note:* Standard length (SL, mm) is reported as minimum–maximum (mean ± SD) for the individuals included in the corresponding dataset.

Morphological identification was conducted based on voucher specimens following taxonomic revisions and regional faunal works (Randall and Kulbicki 2006; Uiblein and Heemstra 2010), using diagnostic meristic and morphometric characters and, where informative, relatively stable coloration and pattern characters.

### 2.2 Morphological Analysis

Morphometric measurements were taken on the left side of each specimen using digital calipers to the nearest 0.01 mm following Uiblein and Heemstra (2010). The dataset comprised 43 linear measurements and eight meristic counts, reported as raw values in Tables 2 and 3.

**TABLE 2:** Morphometric data for *Upeneus* and *Mulloidichthys*.

Morphometric measurements	*M. flavolineatus*	*M. vanicolensis*	*U. heterospinus*	*U. itoui*	*U. japonicus*	*U. sulphureus*	*U. sundaicus*	*U. tragula*
**Number**	5	6	18	5	39	63	8	31
**Counts**								
First dorsal‐fin spines and rays	VIII, 9	VIII, 9	VII–VIII, 9	VII, 9	VII, 9	VIII, 9	VIII, 9	VIII, 9
Anal‐fin spines and rays	I‐6	I‐6	I‐6	I‐7	I‐6	I‐6	I‐7–8	I‐6
Pelvic‐fin rays	I‐5	I‐5	I‐5	I‐5	I‐5	I‐5	I‐5	I‐5
Pectoral‐fin rays	17	17	14	13–15	13	14–17	13–14	13
Lateral‐line scales	34	34–35	—	29–31	27–28	26–36	28–31	27–30
Scales above lateral line	2.5	2.5–3.5	—	2	—	1.5–3	2–2.5	2–29
Scales below lateral line	6	5.5–6	—	4–5	—	3.5–6	4–5	4–5.5
Total gill rakers	7–8 + 17–20	7–9 + 22–24	5–7 + 14–17	5–7 + 15–16	5–8 + 12–18	7–9 + 17–20	4–6 + 13–15	4–8 + 13–17
**Measurements**								
Standard length (SL)	138.6–213.7 (174.3)	145.6–250.3 (218.2)	94.0–130.5 (112.5)	101.1–105.2 (103.2)	89.1–142.9 (104.0)	95.0–150.5 (124.0)	101.5–146.5 (120.3)	88.5–198.5 (133.7)
Body depth at first dorsal fin origin	32.2–49.8 (41.7)	40.8–70.5 (61.0)	22.9–37.7 (29.8)	25.1–27.7 (26.5)	23.6–36.8 (28.2)	27.7–45.1 (37.7)	28.0–40.7 (33.6)	22.1–55.3 (34.4)
Body width	19.2–31.5 (25.5)	22.0–36.4 (32.0)	12.4–19.6 (16.2)	15.0–18.1 (16.1)	8.7–20.5 (13.4)	12.9–23.0 (17.6)	12.4–19.2 (16.2)	9.5–28.5 (17.9)
Body depth at anal‐fin origin	29.2–44.1 (37.0)	37.0–61.2 (53.1)	19.3–33.9 (25.7)	23.3–25.3 (23.9)	19.7–31.5 (23.8)	25.3–39.8 (33.4)	25.0–35.4 (28.9)	19.7–48.7 (30.9)
Half body depth at first dorsal‐fin origin	26.5–39.9 (32.9)	29.3–53.7 (47.2)	18.2–29.9 (23.9)	20.3–23.3 (21.5)	17.1–30.4 (23.2)	21.6–37.9 (29.2)	20.9–33.6 (26.3)	3.9–47.6 (27.4)
Half body depth at anal‐fin origin	21.0–30.5 (25.8)	24.3–44.1 (38.0)	13.4–25.9 (18.6)	14.6–18.6 (16.2)	14.3–24.1 (18.9)	17.8–31.9 (25.2)	16.4–24.8 (19.3)	15.2–35.9 (23.3)
Caudal‐peduncle depth	12.9–20.4 (16.4)	14.8–25.1 (22.1)	9.7–14.7 (12.0)	10.0–11.4 (10.5)	8.7–14.0 (11.1)	10.5–18.5 (14.5)	12.0–18.0 (14.2)	4.2–21.7 (14.4)
Caudal‐peduncle width	6.0–9.6 (7.4)	5.2–10.9 (8.9)	3.2–6.8 (4.5)	4.0–5.0 (4.6)	3.0–6.0 (4.4)	4.2–7.4 (5.7)	4.8–5.9 (5.4)	2.5–8.3 (5.4)
Caudal‐peduncle length	26.1–46.2 (36.7)	30.0–44.2 (40.2)	20.8–29.6 (25.4)	22.1–26.3 (24.3)	17.2–30.0 (21.9)	15.3–27.0 (21.8)	21.8–32.2 (27.3)	20.0–47.2 (29.9)
Barbel length	30.6–42.4 (36.6)	31.1–59.0 (50.8)	14.5–25.0 (18.9)	16.9–19.1 (17.9)	15.8–28.9 (20.6)	11.7–27.0 (19.4)	16.4–25.9 (20.7)	15.8–35.6 (22.5)
Maximum barbel width	1.3–1.9 (1.7)	1.1–3.8 (2.8)	0.9–1.4 (1.1)	1.0–1.5 (1.2)	0.6–1.2 (0.8)	0.7–3.7 (2.1)	0.9–1.3 (1.0)	0.8–2.3 (1.3)
First predorsal length	55.9–79.5 (66.4)	54.8–99.7 (84.5)	31.7–45.6 (37.2)	30.2–37.1 (34.0)	27.1–44.6 (34.3)	32.1–57.4 (43.9)	33.3–50.0 (39.2)	31.1–68.5 (46.5)
Second predorsal length	95.1–146.5 (118.7)	98.1–176.6 (150.4)	61.1–86.6 (72.1)	63.2–68.7 (65.9)	57.6–91.7 (67.6)	63.6–103.1 (84.2)	60.3–94.9 (74.9)	56.9–129.7 (86.4)
Inter dorsal distance	15.4–30.7 (23.0)	15.9–28.9 (21.8)	14.6–19.7 (16.6)	11.4–15.2 (13.5)	11.3–25.5 (17.5)	16.1–26.8 (21.7)	14.9–17.7 (15.6)	8.7–30.8 (19.8)
Caudal‐fin length	44.7–64.6 (53.6)	42.5–66.1 (56.5)	26.6–34.0 (30.6)	25.3–31.0 (28.8)	17.7–31.5 (24.0)	19.7–41.1 (28.2)	28.4–38.4 (32.1)	22.3–54.1 (38.4)
Preanal length	98.5–146.9 (121.2)	100.2–181.7 (156.0)	60.4–86.3 (74.4)	64.3–68.8 (66.4)	57.1–93.3 (69.3)	66.0–107.7 (88.2)	63.0–98.3 (78.0)	58.7–134.2 (88.2)
Prepelvic length	50.0–67.2 (58.1)	46.9–85.8 (72.9)	27.5–38.4 (32.5)	28.4–30.3 (29.3)	23.5–41.5 (29.4)	27.1–49.3 (38.5)	17.3–42.4 (33.3)	27.5–64.9 (41.2)
Prepectoral length	47.3–64.0 (54.7)	44.7–80.9 (68.2)	26.1–36.0 (30.2)	26.9–30.9 (28.5)	22.2–37.5 (28.3)	26.5–46.7 (35.4)	26.7–40.6 (32.2)	26.0–58.4 (37.6)
Second dorsal‐fin depth	30.3–46.1 (37.9)	37.7–64.5 (55.8)	20.6–34.4 (26.6)	23.2–26.0 (24.1)	20.6–33.2 (24.6)	26.0–40.2 (34.4)	22.7–36.2 (28.6)	20.3–49.0 (31.7)
Pelvic‐fin depth	33.0–53.9 (42.6)	41.4–72.4 (62.9)	23.1–37.4 (29.7)	25.5–27.9 (26.5)	24.5–39.6 (29.2)	29.3–47.7 (39.7)	24.9–40.9 (31.2)	22.5–55.5 (34.4)
Pectoral‐fin depth	15.9–33.8 (24.8)	27.3–47.0 (39.7)	13.7–23.4 (18.6)	15.6–18.4 (16.9)	13.2–26.5 (17.8)	19.1–31.4 (25.3)	16.4–26.5 (20.4)	13.1–34.1 (21.3)
Length of first dorsal‐fin base	23.9–36.9 (29.1)	24.7–50.6 (42.6)	12.9–25.6 (18.0)	16.2–18.8 (17.7)	9.8–22.3 (15.1)	11.5–22.8 (17.5)	14.1–28.6 (20.0)	12.4–28.1 (19.0)
Length of second dorsal‐fin base	18.9–26.3 (23.1)	20.8–35.6 (31.6)	13.4–20.1 (16.2)	11.1–16.9 (14.7)	10.5–18.8 (14.4)	11.4–20.5 (16.7)	13.3–22.8 (16.3)	13.2–26.4 (18.5)
Length of pectoral‐fin base	8.8–14.6 (10.3)	7.5–15.4 (12.8)	3.9–8.6 (5.7)	4.9–6.5 (5.7)	3.4–7.6 (4.9)	3.6–7.7 (5.7)	4.6–9.6 (6.7)	3.4–30.9 (6.9)
Length of anal‐fin base	14.3–20.3 (16.6)	16.0–28.4 (24.6)	7.2–16.8 (12.5)	11.2–14.0 (12.2)	6.7–13.8 (10.7)	9.5–15.2 (12.2)	10.1–16.6 (12.8)	9.9–21.8 (13.9)
Length of pelvic‐fin base	5.7–7.3 (6.5)	6.1–9.5 (8.1)	3.3–5.8 (4.5)	5.2–5.5 (5.3)	2.8–5.2 (3.9)	3.1–6.8 (4.6)	4.9–6.5 (5.7)	3.2–8.0 (5.4)
Pelvic‐fin length	27.7–41.7 (34.9)	28.2–48.8 (43.7)	16.9–26.8 (21.8)	14.1–19.0 (17.2)	14.7–22.9 (19.2)	14.1–25.0 (19.5)	19.8–30.0 (23.0)	17.4–37.1 (25.2)
Pectoral‐fin length	27.7–42.4 (35.9)	30.6–59.1 (50.8)	19.5–28.6 (23.1)	17.2–22.1 (20.4)	17.3–29.4 (22.4)	21.5–35.3 (27.8)	20.6–30.4 (24.3)	16.6–36.1 (24.3)
Anal‐fin length	9.7–30.7 (18.3)	9.5–14.4 (11.3)	9.2–18.0 (14.3)	8.1–25.3 (17.3)	13.8–23.2 (18.9)	6.5–17.1 (11.6)	22.9–39.8 (34.4)	14.1–17.3 (15.9)
First dorsal‐fin height	17.0–37.0 (23.3)	22.5–38.8 (27.9)	17.0–31.2 (21.2)	15.2–34.5 (26.9)	25.7–46.7 (36.5)	18.3–27.0 (22.4)	31.4–49.0 (43.4)	18.1–21.6 (19.7)
Second dorsal‐fin height	9.9–29.4 (18.2)	15.0–23.4 (18.7)	8.2–20.6 (13.6)	11.6–21.4 (17.9)	14.3–19.0 (16.8)	10.9–19.5 (16.5)	13.6–24.1 (20.0)	14.2–17.5 (16.0)
Maximum head depth	20.2–50.3 (31.5)	23.5–37.0 (29.2)	21.5–32.7 (25.3)	25.7–40.9 (34.8)	32.0–50.6 (40.5)	23.1–34.0 (27.5)	37.6–65.7 (57.4)	23.0–25.1 (24.0)
Head depth through eye	14.7–35.5 (22.5)	16.0–26.7 (19.9)	13.7–22.4 (17.2)	16.8–29.0 (23.0)	25.8–38.1 (31.6)	15.0–22.5 (18.3)	27.1–49.0 (43.0)	15.9–17.2 (16.4)
Suborbital depth	7.6–21.5 (12.6)	8.3–17.0 (11.7)	7.6–15.7 (11.1)	8.6–20.2 (14.0)	13.9–21.3 (16.8)	6.4–13.0 (9.5)	12.2–26.4 (22.3)	7.2–9.3 (8.5)
Interorbital length	5.7–15.6 (9.9)	6.8–9.5 (8.3)	5.8–10.3 (7.4)	7.0–12.6 (9.8)	10.4–15.6 (13.3)	7.4–10.4 (8.3)	10.3–23.1 (18.2)	7.8–8.2 (8.1)
Head length	25.1–55.4 (36.4)	23.8–38.6 (30.7)	20.9–34.2 (26.1)	23.8–41.4 (31.6)	43.8–59.0 (51.0)	24.0–34.4 (28.4)	43.0–75.9 (64.0)	25.8–28.6 (26.6)
Snout length	6.3–18.2 (10.5)	7.8–12.5 (10.2)	3.3–10.5 (6.5)	3.1–10.6 (6.4)	17.0–22.6 (18.8)	6.4–10.0 (8.1)	15.0–27.1 (20.3)	7.5–10.5 (8.5)
Post orbital length	10.6–24.3 (15.8)	9.7–14.6 (12.0)	8.1–14.4 (11.4)	11.7–19.2 (14.6)	13.9–21.2 (18.0)	9.7–15.3 (11.8)	15.2–30.9 (26.0)	11.1–11.8 (11.5)
Orbit length	7.3–14.8 (10.4)	7.0–11.0 (8.7)	4.8–10.8 (7.9)	6.8–14.4 (10.3)	12.6–16.3 (14.1)	7.0–10.6 (8.7)	12.9–20.2 (17.7)	5.6–6.8 (6.2)
Orbit depth	6.8–13.7 (9.8)	6.0–9.5 (7.2)	5.5–10.6 (7.5)	7.2–13.9 (10.5)	12.0–14.4 (13.3)	5.6–10.3 (7.9)	13.4–20.9 (17.5)	6.1–7.1 (6.7)
Upper‐jaw length	6.7–16.0 (10.7)	7.7–12.6 (10.8)	4.4–11.0 (7.1)	5.6–13.4 (9.6)	13.5–20.0 (15.7)	7.5–14.0 (10.2)	15.0–28.0 (23.4)	6.1–11.2 (9.2)
Lower‐jaw length	5.9–15.6 (9.5)	5.9–11.0 (8.9)	3.8–10.0 (6.3)	5.0–12.1 (8.9)	12.5–19.0 (14.7)	5.8–13.0 (8.9)	14.0–24.5 (21.4)	5.5–9.5 (8.1)
Snout width	6.2–18.1 (10.9)	7.5–10.1 (8.3)	4.6–9.5 (7.0)	5.5–12.7 (9.1)	8.5–13.0 (10.7)	5.8–12.0 (9.3)	10.0–22.0 (18.2)	8.4–9.6 (9.1)

*Note:* —not measured due to specimen damage.

**TABLE 3:** Morphometric data for *Parupeneus*.

Morphometric measurements	*P. barberinoides*	*P. barberinus*	*P. biaculeatus*	*P. chrysopleuron*	*P. ciliatus*	*P. crassilabris*	*P. cyclostomus*	*P. heptacantha*	*P. indicus*	*P. multifasciatus*
**Number**	2	5	32	24	4	2	4	5	16	14
**Counts**										
First dorsal‐fin spines and rays	VIII, 9	VIII, 9	VIII, 9	VIII, 9	VII—VIII, 9	VIII, 9	VIII, 9	VIII, 9	VIII, 9	VIII, 9
Anal‐fin spines and rays	I‐7	I‐7	I‐7	I‐7	I‐7	I‐7	I‐7	I‐7	I‐7	I‐7
Pelvic‐fin rays	I‐5	I‐5	I‐5	I‐5	I‐5	I‐5	I‐5	I‐5	I‐5	I‐5
Pectoral‐fin rays	15	16–17	15–16	15	14–15	15–16	14–15	15–16	15–16	15–16
Lateral‐line scales	27	27–30	26–31	26–29	34–35	27–28	26–28	26–29	26–35	26–31
Scales above lateral line	6 + 20–21	4–7 + 16–21	4–7 + 18–23	4–7 + 13–21	6–7 + 24–25	8 + 28–30	4–6 + 21–23	5–7 + 18–21	5–8 + 12–18	6–10 + 24–30
**Measurements**										
Standard length (SL)	163.1–177.3 (170.2)	268.2–343.3 (303.4)	114.5–217.0 (150.4)	113.3–177.0 (134.7)	132.0–203.4 (178.9)	236.4–250.4 (243.4)	206.6–330.2 (290.1)	129.6–182.0 (161.8)	111.1–247.8 (188.1)	119.5–189.1 (152.7)
Body depth at first dorsal‐fin origin	54.5–58.9 (56.7)	76.0–106.9 (89.3)	37.1–74.8 (49.8)	39.0–56.8 (45.1)	45.1–69.4 (62.2)	83.2–94.5 (88.9)	55.0–98.0 (85.7)	41.5–60.2 (53.1)	32.9–83.6 (59.4)	36.6–63.9 (49.5)
Body width	25.2–25.4 (25.3)	37.9–52.6 (44.0)	16.9–33.0 (22.8)	15.5–24.9 (19.8)	22.6–33.6 (30.8)	35.8–39.0 (37.4)	30.5–48.3 (42.4)	20.3–30.6 (25.3)	15.0–44.0 (30.0)	16.4–29.6 (21.5)
Body depth at anal‐fin origin	46.4–52.5 (49.5)	62.3–94.4 (77.6)	33.8–69.3 (44.7)	32.3–48.2 (39.5)	40.1–64.7 (56.1)	68.9–80.7 (74.8)	54.7–90.3 (77.4)	35.4–52.7 (44.5)	11.1–73.5 (49.9)	33.1–55.0 (44.4)
Half body depth at first dorsal‐fin origin	46.1–49.2 (47.7)	61.9–84.4 (72.5)	29.2–59.4 (39.2)	32.6–45.4 (37.1)	35.0–56.5 (50.8)	65.2–71.2 (68.2)	46.9–78.0 (68.3)	28.0–49.2 (41.7)	0.6–69.0 (44.2)	26.8–51.6 (38.8)
Half body depth at anal‐fin origin	35.5–40.2 (37.9)	45.5–70.9 (56.4)	23.9–50.5 (32.8)	25.4–39.1 (30.1)	28.4–47.1 (41.5)	50.2–57.6 (53.9)	39.5–63.5 (56.3)	26.7–38.7 (32.9)	22.3–53.4 (38.7)	23.3–41.3 (32.4)
Caudal‐peduncle depth	19.8–23.8 (21.8)	31.0–40.4 (35.5)	15.0–28.3 (19.2)	13.1–20.4 (15.9)	17.6–27.6 (24.2)	31.8–32.5 (32.2)	24.1–41.2 (35.8)	14.3–19.7 (18.0)	13.1–35.8 (24.2)	13.7–23.2 (18.6)
Caudal‐peduncle width	7.2–7.2 (7.2)	9.9–14.1 (12.2)	4.7–9.9 (7.2)	4.3–8.7 (5.7)	6.7–8.5 (8.0)	8.6–10.1 (9.4)	9.4–12.7 (11.1)	5.4–9.5 (6.7)	5.2–13.7 (9.0)	5.7–10.0 (7.4)
Caudal‐peduncle length	33.3–41.7 (37.5)	53.5–65.5 (58.3)	24.2–45.0 (32.5)	20.8–38.8 (27.4)	28.8–43.1 (37.8)	46.8–48.2 (47.5)	39.2–58.6 (53.2)	26.8–43.2 (36.4)	25.8–51.6 (38.8)	20.7–43.9 (28.3)
Barbel length	46.4–47.3 (46.9)	59.7–86.0 (69.6)	8.2–46.0 (33.9)	25.3–38.6 (30.2)	26.9–39.1 (34.1)	47.4–56.0 (51.7)	64.2–101.1 (89.1)	33.7–47.9 (42.0)	25.0–56.0 (42.4)	30.4–58.0 (41.9)
Maximum barbel width	2.3–2.7 (2.5)	3.5–4.3 (3.9)	1.2–2.4 (1.7)	1.1–1.8 (1.5)	1.2–2.1 (1.9)	2.4–2.9 (2.7)	2.3–3.6 (3.1)	1.7–2.2 (2.0)	1.1–3.7 (2.4)	1.4–2.6 (2.0)
First predorsal length	60.1–62.3 (61.2)	93.1–132.0 (116.2)	41.7–75.5 (52.5)	39.4–62.4 (46.0)	49.2–76.6 (64.4)	90.0–100.2 (95.1)	72.2–133.8 (110.0)	47.3–65.7 (57.1)	43.1–91.4 (68.5)	46.8–72.6 (60.1)
Second predorsal length	104.1–111.9 (108.0)	172.2–226.4 (198.9)	74.3–139.2 (95.1)	73.1–113.2 (85.8)	86.4–134.5 (116.4)	147.6–162.9 (155.3)	130.1–218.6 (186.8)	84.8–115.7 (102.5)	71.3–159.0 (119.8)	77.1–118.4 (98.6)
Inter dorsal distance	21.1–24.5 (22.8)	20.0–27.8 (23.6)	8.0–30.5 (14.4)	8.3–20.9 (11.4)	15.4–24.6 (21.6)	11.6–13.8 (12.7)	15.0–32.5 (24.0)	13.4–21.4 (19.3)	14.5–30.9 (23.9)	6.5–19.2 (15.2)
Caudal‐fin length	43.6–44.7 (44.2)	78.2–126.1 (90.6)	30.4–60.5 (41.0)	27.7–52.7 (36.3)	33.7–48.8 (43.5)	63.2–70.5 (66.9)	61.3–88.2 (74.7)	34.5–52.8 (47.9)	33.8–75.6 (54.1)	24.3–52.7 (39.9)
Preanal length	104.0–111.1 (107.6)	181.4–231.8 (213.6)	79.3–139.2 (97.7)	9.6–114.9 (83.7)	88.9–136.9 (119.0)	158.9–173.3 (166.1)	204.9–221.0 (213.7)	100.4–118.9 (111.2)	73.9–162.7 (123.3)	77.6–120.6 (99.2)
Prepelvic length	53.6–60.1 (56.9)	85.1–113.8 (101.1)	36.8–68.1 (46.2)	34.6–56.5 (41.0)	42.2–65.3 (56.3)	75.8–85.2 (80.5)	68.2–109.5 (93.4)	38.7–55.5 (47.9)	33.6–89.6 (60.7)	37.5–59.9 (50.7)
Prepectoral length	55.3–61.5 (58.4)	82.1–115.6 (102.1)	37.7–68.7 (47.2)	34.7–56.3 (39.8)	45.2–63.4 (55.7)	76.0–83.9 (80.0)	73.5–113.9 (96.1)	40.2–56.8 (49.9)	35.0–85.4 (58.4)	37.8–62.5 (51.8)
Second dorsal‐fin depth	47.5–52.1 (49.8)	70.2–96.8 (81.3)	35.0–69.1 (45.3)	33.5–49.5 (40.2)	41.8–64.8 (57.0)	74.6–84.6 (79.6)	54.6–93.2 (81.3)	35.4–53.9 (45.4)	30.3–74.0 (53.5)	33.7–56.3 (44.7)
Pelvic‐fin depth	54.1–57.0 (55.6)	80.3–112.3 (92.9)	36.5–73.7 (49.3)	38.9–55.6 (45.3)	45.3–69.8 (61.3)	82.7–96.2 (89.5)	59.3–99.5 (88.3)	41.8–60.0 (52.0)	32.9–85.7 (61.5)	37.2–64.7 (51.4)
Pectoral‐fin depth	29.3–31.8 (30.6)	52.1–72.6 (61.4)	22.0–43.1 (29.8)	21.6–35.1 (26.8)	27.1–39.0 (35.6)	49.0–57.7 (53.4)	32.2–60.9 (51.5)	24.3–35.6 (30.4)	19.3–51.1 (37.8)	23.0–37.7 (30.0)
Length of first dorsal‐fin base	24.9–29.2 (27.0)	50.0–58.9 (55.0)	23.7–34.7 (29.1)	24.0–46.6 (29.9)	21.3–34.6 (30.0)	45.7–51.2 (48.5)	35.2–60.4 (51.6)	23.5–30.5 (27.0)	16.4–37.3 (27.3)	17.2–34.8 (24.0)
Length of second dorsal‐fin base	27.0–31.4 (29.2)	44.1–64.0 (53.5)	21.1–37.9 (27.6)	20.0–37.5 (25.5)	20.8–34.6 (30.5)	46.3–52.2 (49.3)	33.2–64.3 (52.6)	21.7–30.6 (25.9)	12.9–41.7 (28.1)	19.0–35.4 (26.3)
Length of pectoral‐fin base	10.0–11.8 (10.9)	18.2–28.2 (21.4)	5.5–12.9 (9.4)	7.6–11.4 (9.9)	7.5–12.1 (10.5)	15.5–17.9 (16.7)	11.3–20.2 (17.1)	8.1–11.5 (9.6)	6.3–16.8 (11.2)	5.5–15.5 (8.5)
Length of anal‐fin base	23.5–25.2 (24.3)	36.8–47.3 (41.0)	15.8–27.6 (20.9)	17.0–25.0 (19.8)	15.8–22.5 (19.6)	30.3–36.4 (33.4)	26.8–49.2 (41.8)	16.9–23.3 (19.8)	13.4–31.8 (23.0)	13.6–31.0 (21.5)
Length of pelvic‐fin base	7.6–7.7 (7.7)	11.3–15.9 (13.0)	4.7–9.1 (6.5)	4.7–7.9 (6.1)	7.0–10.1 (8.9)	10.5–12.5 (11.5)	10.4–14.4 (12.9)	5.4–9.4 (7.2)	4.7–12.7 (9.2)	4.2–10.0 (6.7)
Pelvic‐fin length	45.7–46.8 (46.2)	58.2–74.0 (66.2)	25.7–42.1 (31.6)	25.0–33.0 (29.5)	31.6–45.5 (41.9)	59.0–66.2 (62.6)	43.2–68.2 (58.7)	27.4–36.3 (33.2)	26.0–48.6 (34.6)	28.8–49.1 (38.5)
Pectoral‐fin length	42.6–45.4 (44.0)	54.6–80.7 (69.1)	30.4–43.9 (36.6)	30.4–43.6 (34.3)	28.6–39.9 (35.2)	66.0–67.6 (66.8)	40.9–78.0 (66.7)	30.0–42.2 (37.2)	24.5–50.9 (38.2)	20.0–46.6 (33.8)
Anal‐fin length	16.3–25.5 (20.9)	38.1–79.0 (58.4)	12.4–30.6 (20.4)	10.5–19.7 (12.6)	18.3–30.8 (24.5)	54.0–62.1 (58.1)	28.3–79.2 (64.3)	14.6–24.9 (18.6)	17.3–34.4 (23.9)	11.6–23.7 (17.8)
First dorsal‐fin height	26.2–30.2 (28.2)	78.2–121.2 (103.6)	21.8–45.2 (28.9)	19.0–30.3 (23.3)	20.6–39.2 (33.6)	39.4–61.6 (50.5)	40.4–81.0 (68.0)	20.3–39.8 (26.9)	21.5–62.2 (40.2)	20.7–50.6 (34.1)
Second dorsal‐fin height	15.6–16.4 (16.0)	32.0–43.2 (38.1)	13.5–26.0 (19.8)	7.5–15.3 (11.2)	15.1–27.6 (21.1)	25.0–30.0 (27.5)	26.4–34.3 (31.6)	12.5–20.3 (16.0)	13.8–33.2 (22.9)	10.5–20.4 (14.6)
Maximum head depth	52.5–58.7 (55.6)	76.1–105.6 (88.1)	34.7–73.3 (46.5)	36.5–54.9 (42.6)	43.0–63.7 (57.7)	80.9–91.3 (86.1)	55.2–95.5 (83.6)	39.8–58.1 (49.8)	31.7–80.2 (57.9)	34.8–62.3 (48.0)
Head depth through eye	42.0–45.1 (43.6)	61.2–82.7 (70.6)	26.7–51.4 (33.5)	26.8–45.0 (31.4)	29.5–46.6 (40.8)	66.2–70.3 (68.3)	49.0–80.4 (70.3)	28.4–45.0 (37.9)	25.2–62.7 (44.0)	26.6–49.2 (37.4)
Suborbital depth	27.6–28.6 (28.1)	40.0–60.9 (49.9)	13.8–33.6 (20.0)	17.0–28.3 (20.7)	16.6–28.7 (24.9)	41.8–44.1 (43.0)	31.5–55.0 (47.8)	17.2–28.7 (23.5)	16.8–44.9 (30.8)	16.9–33.3 (24.4)
Interorbital length	15.9–18.0 (16.9)	19.8–24.7 (21.8)	8.1–18.5 (12.8)	8.7–14.1 (10.4)	13.1–21.6 (18.8)	23.1–24.5 (23.8)	17.0–28.6 (24.8)	9.6–15.7 (13.6)	9.8–27.0 (18.0)	10.2–18.0 (13.4)
Head length	52.1–59.3 (55.7)	83.9–114.0 (101.9)	36.5–66.2 (45.0)	31.8–55.4 (38.2)	43.5–59.0 (52.9)	75.5–79.4 (77.5)	69.2–113.7 (93.2)	38.5–56.6 (48.7)	34.5–81.7 (56.7)	36.9–60.4 (49.8)
Snout length	25.5–26.9 (26.2)	37.1–57.7 (49.7)	12.6–30.8 (17.6)	10.6–23.8 (13.8)	16.2–24.1 (20.5)	32.5–36.0 (34.3)	37.0–58.5 (46.5)	14.7–23.9 (20.5)	13.6–38.1 (22.8)	14.7–30.2 (22.9)
Post orbital length	16.5–21.6 (19.1)	32.6–43.6 (37.8)	13.8–23.4 (17.0)	9.9–18.6 (14.0)	17.0–22.6 (20.2)	26.6–31.6 (29.1)	22.8–42.7 (34.9)	14.1–19.7 (17.2)	13.0–27.6 (21.5)	10.4–22.9 (15.6)
Orbit length	10.5–10.9 (10.7)	12.9–18.0 (14.6)	8.5–12.9 (10.6)	8.5–12.8 (10.6)	10.9–13.4 (12.3)	14.0–15.7 (14.9)	10.2–14.9 (12.0)	10.9–13.0 (12.1)	7.2–16.4 (11.7)	8.2–11.3 (9.8)
Orbit depth	9.3–10.9 (10.1)	11.3–16.2 (13.8)	7.8–11.6 (10.3)	7.8–11.3 (9.8)	9.3–13.9 (11.9)	13.8–17.8 (15.8)	9.3–13.6 (11.7)	8.8–12.6 (10.3)	6.2–17.5 (11.7)	7.6–12.0 (10.0)
Upper‐jaw length	11.7–11.9 (11.8)	24.0–35.0 (29.8)	9.1–18.0 (13.6)	12.0–18.2 (13.7)	9.3–12.6 (10.6)	33.0–34.0 (33.5)	26.0–41.0 (36.1)	14.9–20.3 (17.4)	5.5–22.8 (14.9)	17.2–27.0 (22.7)
Lower‐jaw length	8.4–11.1 (9.7)	22.5–33.4 (27.4)	6.9–17.0 (12.2)	11.0–17.0 (12.7)	7.7–9.4 (8.5)	28.5–30.0 (29.3)	22.2–38.5 (32.3)	10.2–19.4 (15.4)	4.2–19.6 (12.4)	16.2–26.0 (21.7)
Snout width	11.0–12.1 (11.6)	15.5–27.8 (21.6)	7.8–19.6 (11.1)	7.4–11.8 (9.4)	10.3–16.9 (14.7)	22.6–22.9 (22.8)	12.2–28.8 (24.0)	9.3–16.6 (13.0)	7.6–26.1 (16.3)	6.2–112.0 (16.5)

*Note:* — not measured due to specimen damage.

To reduce the confounding influence of body size on linear morphometric traits, measurements were size‐adjusted using the allometric method of Elliott et al. (1995), as follows:

$$M_{\mathrm{adj}}=M{\left({\mathrm{SL}}_{s}/{\mathrm{SL}}_{0}\right)}^{b}$$

where *M*_*adj*_ is the size‐adjusted measurement, *M* is the original measurement, *SL*_s_ is a reference standard length (set to the median SL of all specimens included in the analysis), and *SL*_0_ is the standard length of each individual. For each trait, the allometric exponent *b* was estimated as the slope from a log–log regression of the trait on standard length (SL), log(*M*) = *a* + *b* log(*SL*). The effectiveness of size adjustment was evaluated by examining residual size dependence via correlations between *M*_*adj*_ and SL. Because specimens were not dissected, sex and sexual maturity could not be determined and were therefore not evaluated as sources of morphometric variation.

All multivariate analyses were conducted using the size‐adjusted and standardized morphometric variables described above. After size adjustment, linear measurements were log‐transformed and z‐standardized. Principal component analysis (PCA) was used to summarize multivariate variation and visualize specimens in morphospace. For hypothesis testing in multivariate space, Euclidean distance matrices (used for PERMANOVA and dispersion tests) were computed from the standardized morphometric variables. Genus‐level differences were tested using PERMANOVA (adonis2; 9999 permutations) (Anderson 2001; McArdle and Anderson 2001), and homogeneity of multivariate dispersions among groups was assessed using betadisper (permutest; 9999 permutations) to aid interpretation of PERMANOVA results. Specimens with missing values in any morphometric variable used were excluded (complete‐case analysis). To address heterogeneous size ranges among genera, we repeated the same workflow on a size‐restricted subset defined by the common SL overlap across genera, where the overlap interval was defined as the maximum of genus‐specific minimum SL values and the minimum of genus‐specific maximum SL values, thereby reducing potential confounding associated with heterogeneous size structure among genera. All analyses were performed in R 4.4.2 using the packages vegan, dplyr, ggplot2, and tidyr.

### 2.3 DNA Extraction, PCR Amplification, and Sequencing

After morphometric measurements and photography, approximately 50 mg of dorsal muscle tissue was excised from each specimen, preserved immediately in 95% ethanol, and stored at −20°C until DNA extraction.

Genomic DNA was extracted using the FastPure Cell/Tissue DNA Isolation Mini Kit. Polymerase chain reaction (PCR) amplification was performed to target fragments of the COI, 16S rRNA, and IRBP genes. The following primer pairs were used: FishF1 (forward) and FishR1 (reverse) for COI (Ward et al. 2005), 16Sbr‐F (forward) and 16Sbr‐R (reverse) for 16S rRNA (López et al. 2000), and IRBP U110 and IRBP L936 for IRBP (Dettaï and Lecointre 2008). Primer sequences are detailed in Table S1.

PCR reactions were performed in a total volume of 25 μL, containing 1× Taq PCR Mix (12.5 μL of 2× mix), forward and reverse primers at a final concentration of 0.4 μM each (10 μM working solutions; 1 μL each), and additional MgCl_2_ at a final concentration of 2.0 mM (25 mM stock; 2 μL per reaction), with nuclease‐free water added to volume. Genomic DNA was added at 2 μL per reaction; DNA concentration was not quantified or standardized prior to PCR, and amplification success was verified by agarose gel electrophoresis. Thermal cycling conditions were: initial denaturation at 95°C for 3 min; followed by 35 cycles of denaturation at 92°C for 45 s, annealing at 50°C (for COI and 16S rRNA) or 55°C (for IRBP) for 45 s, and extension at 72°C for 1 min; with a final extension at 72°C for 10 min. PCR products were visualized on 1% agarose gels stained with ethidium bromide, and amplicons with clear single bands were purified and bidirectionally sequenced by Sangon Biotech (Shanghai) Co. Ltd.

### 2.4 Sequence Analysis and Phylogenetic Tree Reconstruction

Forward and reverse Sanger reads were assembled into consensus sequences in Sequencher v5.4.5. Low‐quality ends were trimmed automatically, primer sequences were removed, and base calls were checked against chromatograms, with manual edits applied when necessary. Preliminary COI‐based taxonomic assignments were evaluated using NCBI BLASTn searches against the GenBank nucleotide database. BLAST results were used as an auxiliary validation step to assess congruence with morphological identifications rather than as a standalone criterion for final species assignment; matches to our newly submitted sequences were not treated as independent validation. Specimens showing non‐unique best hits or discordant species names were treated as ambiguous and were further evaluated by re‐examining voucher morphology and by comparison with phylogenetic and species‐delimitation results.

Phylogenetic analysis incorporated COI sequences from three genera and 16 species of Mullidae obtained from GenBank (Table S2). Sequence alignment and trimming were performed in MEGA v11.0 (Tamura et al. 2021), and intraspecific and interspecific genetic distances were calculated using the Kimura two‐parameter (K2P) model.

Outgroup selection adhered to the principle of using closely related taxa (Ware et al. 2008; Luo et al. 2010). Recent phylogenetic studies indicate that Mullidae is closely related to Callionymidae and Draconettidae (Longo et al. 2017; Santaquiteria et al. 2021; Nash et al. 2022). Accordingly, *Draconetta xenica* and *Foetorepus agassizii* were used as preferred outgroups to root the COI and 16S rRNA analyses. To reduce reliance on any single outgroup and to assess rooting robustness in single‐locus mitochondrial trees, *Taractes asper* (Bramidae) was additionally included as an external reference taxon because COI and 16S rRNA sequences are available for this species. Given the limited availability of IRBP sequences for Callionymidae and Draconettidae in GenBank, *Callionymus maculatus* was selected as the outgroup for the IRBP analysis. GenBank accession numbers for all outgroup sequences are provided in Table S2.

Single‐locus phylogenetic analyses were conducted for COI, 16S rRNA, and IRBP. Because COI and 16S rRNA are both mitochondrial markers, these two loci were concatenated to increase the number of informative sites, thereby improving resolution and nodal support at the species level (Rokas et al. 2003; John and Bryan 2013). The mitochondrial sequences were concatenated in PhyloSuite (Zhang et al. 2020). IRBP is a nuclear gene that provides independent genetic evidence; given that mitochondrial and nuclear loci often exhibit discordant evolutionary histories, IRBP was analyzed separately to avoid conflating signals from different histories, and the resulting nuclear phylogeny was used to assess congruence with, and to corroborate, the mitochondrial results (Ballard and Whitlock 2004). Phylogenetic trees were reconstructed using both maximum likelihood (ML) and Bayesian inference (BI). ML analyses were conducted in PhyloSuite (Zhang et al. 2020) using IQ‐TREE (Nguyen et al. 2015). Branch support was evaluated with 50,000 ultrafast bootstrap replicates and the SH‐aLRT test with 1000 replicates (Guindon et al. 2010). The best‐fit substitution model for each gene partition was selected using ModelFinder: TPM2u + F + I + G4 for COI, TPM2u + F + R3 for 16S rRNA, and TN + F + G4 for IRBP. BI analyses were performed in PhyloSuite (Zhang et al. 2020) using MrBayes (Ronquist et al. 2012), with Markov chain Monte Carlo (MCMC) executed for 20,000,000 generations. For BI, the optimal model for each gene partition was also selected using ModelFinder: HKY + F + I + G4 for COI, GTR + F + G4 for 16S rRNA, and HKY + F + G4 for IRBP.

### 2.5 Species Delimitation

Because uneven sampling and interspecific differences in effective population size may bias species boundaries inferred from a single method, we applied two complementary molecular species‐delimitation approaches: ABGD (Automatic Barcode Gap Discovery) and bPTP (Bayesian Poisson Tree Processes). ABGD identifies candidate species partitions by detecting barcode gaps from the distribution of pairwise genetic distances among COI sequences, whereas bPTP infers species boundaries on a phylogram (i.e., a tree with branch lengths) under a Bayesian Poisson Tree Processes model. Species delimitation was performed on the COI dataset only, because 16S rRNA is more conserved and was used here primarily to corroborate COI‐inferred groupings in phylogenetic analyses rather than for standalone delimitation.

ABGD analyses were performed on the ABGD platform using the trimmed and aligned COI dataset (outgroup sequences were excluded prior to ABGD). Pairwise genetic distances were computed under the Kimura two‐parameter model (K80) with TS/TV = 2.0. We used P_min_ = 0.001, P_max_ = 0.1, and Steps = 10. To evaluate the impact of the relative gap width (*X*) on delimitation outcomes, we conducted a sensitivity analysis across *X* = 0.5, 0.8, 1.0, and 1.2, while keeping all other parameters at their default settings (Puillandre et al. 2012). ABGD provides both initial and recursive partitions; we report the more conservative initial partitions as our primary results. bPTP analyses were conducted via the online bPTP server (https://species.h‐its.org/ptp/) with the ML tree as input, and all other settings were kept at their default values (Zhang et al. 2013).

## 3 Results

### 3.1 Morphological Analysis

Based on morphological characteristics, 283 goatfish specimens collected from the NSCS comprised 18 species in 3 genera.

#### 3.1.1 Diagnostic Characters of Mullidae

##### 3.1.1.1 *Upeneus* Cuvier, 1829

Body elongate and slightly laterally compressed. Dorsal and ventral profiles slightly and similarly convex. Head moderately sized; snout blunt. Eye relatively small, positioned superolaterally, slightly closer to the posterodorsal margin of the opercle than to the snout tip. Mouth small; upper jaw slightly longer than lower jaw. Villiform teeth present on the vomer, palatines, and both jaws. A pair of barbels present on the chin. Two separate dorsal fins; first dorsal fin slightly higher than the second dorsal fin. Pectoral fin inserted posterior to the opercle. Pelvic‐fin origin below the pectoral‐fin base and slightly anterior to the first dorsal‐fin origin. Anal fin opposite the second dorsal fin and similar in shape. Caudal fin deeply forked. Body covered with deciduous ctenoid scales. Lateral line complete, running parallel to the dorsal profile and extending to the caudal‐fin base.

###### 3.1.1.1.1 Upeneus japonicus

Dorsal fins VII, 9; anal fin I‐6; pectoral fin 13; pelvic fin I‐5; gill rakers 5–8 + 12–18 (Table 2). Size of specimens examined: SL 89.1–142.9 mm (*n* = 39). Measurements as %SL: body depth 24–31, head length (HL) 21–30. Measurements as %HL: snout length 19–33, orbit diameter 26–38, barbel length 69–100. First dorsal‐fin spine longest. In fresh coloration, dorsum light reddish and venter white; barbels yellow; body without lateral stripes or spots. Both dorsal fins with two light‐red stripes, fin membranes hyaline; pectoral fins light red and almost hyaline; pelvic fins light red; anal‐fin spine distinctly white and the fin membrane hyaline. Caudal fin with 3–5 oblique light‐red bands on upper lobe; lower lobe red with a black band along the distal margin.

*Distribution*: Western Pacific, from western Malaysia and the Philippines northward to Korea and Peter the Great Bay, Russia. In China, the northern and southern South China Sea, the Beibu Gulf, waters off Taiwan, the East China Sea, and the Yellow and Bohai Seas.

###### 3.1.1.1.2 Upeneus sundaicus

Dorsal fins VIII, 9; anal fin I‐7–8; pectoral fin 13–14; pelvic fin I‐5; gill rakers 4–6 + 13–15 (Table 2). Size of specimens examined: SL 101.5–146.5 mm (*n* = 8). Measurements as %SL: body depth 25–31, head length 22–29. Measurements as %HL: snout length 29–36, orbit length 21–29, barbel length 63–77. First dorsal‐fin spine very short. In fresh coloration, dorsum light brown; lower body white, belly yellow; barbels yellow. A brownish longitudinal stripe present on the side of the body, extending from behind the eye to the caudal‐fin base and running parallel to the lateral line. No spots are present on the head or body side. Dorsal, pectoral, pelvic, and anal fins are light red, without oblique bands. Caudal fin with upper lobe red and lower lobe dark red; upper lobe with several indistinct oblique bands or none, lower lobe without oblique bands.

*Distribution*: Western Pacific, from Pakistan, India, and Sri Lanka eastward to Indonesia, and from northwestern Australia north to Japan. In China, the northern South China Sea, the Beibu Gulf, and waters off Taiwan.

###### 3.1.1.1.3 Upeneus sulphureus

Dorsal fins VIII, 9; anal fin I‐6; pectoral fin 14–17; pelvic fin I‐5; gill rakers 7–9 + 17–20 (Table 2). Size of specimens examined: SL 95.0–150.5 mm (*n* = 63). Measurements as %SL: body depth 27–33, head length 21–31. Measurements as %HL: snout length 17–30, orbit length 26–45, barbel length 40–83. First spine of the first dorsal fin very short; third spine the longest. In fresh coloration, dorsum light red, lower body silvery white, belly yellow; barbels white. Two yellow longitudinal stripes are present on the side of the body, the upper stripe extending from the opercle to the caudal‐fin base, and the lower stripe situated below it. In preserved specimens, the upper stripe usually remains distinct, whereas the lower stripe becomes faint. No spots are present on the side of the body. Each dorsal fin bears 2–3 yellow oblique bands; the first dorsal fin has a large black blotch at the tip. Pectoral fin light red and nearly hyaline; pelvic and anal fins pale yellow. Caudal fin light red, without oblique bands; the outer upper margin of the lower lobe white, immediately followed by a yellow marginal band.

*Distribution*: Indo‐West Pacific, from East Africa to Southeast Asia, north to China, and south to northern Australia and Fiji. In China, the northern and southern South China Sea, the Beibu Gulf, waters off Taiwan, and the East China Sea.

###### 3.1.1.1.4 Upeneus tragula

Dorsal fins VIII, 9; anal fin I‐6; pectoral fin 13; pelvic fin I‐5; gill rakers 4–8 + 13–17 (Table 2). Size of specimens examined: SL 88.5–198.5 mm (*n* = 31). Measurements as %SL: body depth 22–29, head length 24–31. Measurements as %HL: snout length 23–36, orbit length 22–33, barbel length 50–71. First spine of the first dorsal fin very short; third spine the longest. In fresh coloration, dorsum dark purple, venter pale yellow; barbels orange. A black longitudinal stripe is present on the side of the body, extending from behind the eye to the caudal‐fin base. Head and the side of the body have irregular black blotches. First dorsal fin with a large black blotch at the tip; second dorsal fin with oblique black bands; membranes of both dorsal fins pale yellow. Pelvic and anal fins pale yellow with irregular black blotches. Caudal fin pale yellow; upper and lower lobes each with 5–6 oblique bands, with the distal bands on the lower lobe being broader.

*Distribution*: Eastern Indian Ocean to the western Pacific, from the Andaman Islands east to eastern Australia and New Caledonia, and north to Japan. In China, the northern and southern South China Sea, the Beibu Gulf, waters off Taiwan, and the East China Sea.

###### 3.1.1.1.5 Upeneus heterospinus

Dorsal fins VII, 9; anal fin I‐6; pectoral fin 14; pelvic fin I‐5; gill rakers 4–6 + 15–18 (Table 2). Size of specimens examined: SL 94.0–130.5 mm (*n* = 18). Measurements as %SL: body depth 24–29, head length 21–29. Measurements as %HL: snout length 23–34, orbit length 26–34, barbel length 56–83. First dorsal‐fin spine the longest. Head and dorsum red and venter white, usually with faint beige or gray spots or red blotches. Caudal fin with 3–4 red or brown bands or brown bands on the upper lobe and 3–5 red, brown, or dark‐brown bands on the lower lobe, the latter commonly fully or partly traversed by yellow, beige, pale‐red, or pale‐brown bands; the tip of the lower lobe sometimes black. Barbels yellow. A single yellow, beige, or pale‐brown longitudinal stripe present on the side of the body from the snout tip through the eye to the caudal‐fin base, approximately equal to the orbit diameter in width; this stripe bears 1–4 black spots, and the posterior‐most spot lies just behind the base of the second dorsal fin and is usually continuous with a dark postdorsal saddle. The first dorsal fin with 3–4 usually confluent pale‐red, pale‐brown, or beige stripes, without darker pigmentation distally; the second dorsal fin with 3–4 red or brown stripes separated by distinct hyaline interspaces. Pectoral fin hyaline. Pelvic and anal fins weakly pigmented and partially hyaline.

*Distribution*: Western Pacific, from southern Indonesia to Singapore and the central Philippines, extending to the Gulf of Thailand, Vietnam, southern China, and southern Japan. In China, the northern South China Sea, the Beibu Gulf, and waters off Taiwan.

###### 3.1.1.1.6 Upeneus itoui

Dorsal fins VII, 9; anal fin I‐7; pectoral fin 13–15 (usually 14); pelvic fin I‐5; gill rakers 5–7 + 15–16 (Table 2). Size of specimens examined: SL 101.1–105.2 mm (*n* = 5). Measurements as %SL: body depth 21–25, head depth 18–20. Measurements as %HL: barbel width 6–8. First dorsal‐fin spine the longest. In live and fresh specimens, a reddish‐brown longitudinal stripe is present on the side of the body, extending from the snout tip through the eye to the caudal‐fin base. Barbels white. The first dorsal fin with four irregular white transverse bands and 4–6 reddish‐brown transverse bands, which become dark in preserved specimens. Upper caudal‐fin lobe with 5–7 reddish‐brown transverse bands and 5–9 irregular pure‐white bands; lower lobe with 5–9 short red transverse bands and 5–9 short white bands or spots along the ventral margin; the red or brown bands darken in preserved specimens.

*Distribution*: Northwestern Pacific, southern Japan. In China, the northern South China Sea and the Beibu Gulf.

##### 3.1.1.2 *Parupeneus* Bleeker, 1863

Body elongate and somewhat oval in outline, slightly laterally compressed. Dorsal profile more strongly arched than ventral profile. Head moderately large, with a prominent nuchal hump; snout long. Eye small, positioned superolaterally, closer to the upper posterior margin of the opercle than to the snout tip. Mouth small and subterminal; upper jaw slightly longer than lower jaw. Vomer and palatines edentulous; both jaws with a single row of large, blunt teeth. A pair of barbels present on the chin. Two separate dorsal fins; first dorsal fin markedly higher than the second dorsal fin. Pectoral fin inserted posterior to the opercle. Pelvic‐fin origin below the pectoral‐fin base. Anal fin opposite the second dorsal fin. Caudal fin deeply forked. Body covered with large ctenoid scales. Lateral line complete, running parallel to the dorsal profile and extending to the caudal‐fin base.

###### 3.1.1.2.1 Parupeneus multifasciatus

Dorsal fins VIII, 9; anal fin I‐7; pectoral fin 15–16; pelvic fin I‐5; gill rakers 6–10 + 24–30 (Table 3). Size of specimens examined: SL 119.5–189.1 mm (*n* = 14). Measurements as %SL: body depth 30–34, head length 29–37. Measurements as %HL: snout length 40–53, orbit length 14–27, barbel length 71–100. The last soft ray of the second dorsal fin is produced into a filament, reaching the caudal‐fin base. Body silvery white or pale red. Head with three blue stripes running through the eye to the posterior margin of the opercle; dorsal part of the orbit with scattered blue streaks. Posterior margins of scales reddish brown. The side of the body bears five black transverse bars, located respectively (i) on the anteroventral region before the first dorsal fin, (ii) below the first dorsal fin, (iii) on the ventral region between the first and second dorsal fins, (iv) below the second dorsal fin, and (v) on the caudal peduncle; the posterior two bars are distinct. A black longitudinal stripe extends from the snout through the eye to the posterior end of the second dorsal fin. A large round black blotch is present at the midlateral caudal peduncle. Barbels pale reddish brown. All fins reddish brown; with the first dorsal, pelvic, and caudal fin darker; pelvic and caudal fin with several yellow fin rays. Second dorsal fin and anal fin paler, each with 4–5 horizontal yellow stripes.

*Distribution*: Pacific Ocean, from Christmas Island in the Indian Ocean to the Hawaiian Archipelago, Line Islands, Marquesas, and Tuamotu Archipelago, north to southern Japan, and south to Lord Howe Island and the Rapa Islands. In China, the northern and southern South China Sea, the Beibu Gulf, waters off Taiwan, and the East China Sea.

###### 3.1.1.2.2 Parupeneus crassilabris

Dorsal fins VIII, 9; anal fin I‐7; pectoral fin 14–15; pelvic fin I‐5; gill rakers 8 + 28–30 (Table 3). Size of specimens examined: SL 236.4–250.4 mm (*n* = 2). Measurements as %SL: body depth 36–38, head length 31–32. Measurements as %HL: snout length 43–45, orbit length 18–21, barbel length 63–71. Distal tip of the last ray of the second dorsal fin is elongate, reaching the caudal‐fin base. In fresh coloration, body white. Scale margins are yellow, and the central area of some scales is also yellow. Two elongate oval black blotches are present on the side of the body; the first is located below the midportion of the first dorsal fin and the second below the anterior to middle portion of the second dorsal fin; neither extends ventrally to the belly. A large black blotch on the head is situated posterior to the eye, partially covering the eye. Irregular blue streaks are present around the eye. Second dorsal fin and anal fin have yellow stripes. Caudal fin has yellow horizontal stripes.

*Distribution*: Eastern Indian Ocean and western Pacific, east to Fiji, Tonga, and the Caroline Islands. In China, the northern and southern South China Sea.

###### 3.1.1.2.3 Parupeneus barberinoides

Dorsal fins VIII, 9; anal fin I‐7; pectoral fin 15; pelvic fin I‐5; gill rakers 6 + 20–21 (Table 3). Size of specimens examined: SL 163.1–177.3 mm (*n* = 2). Measurements as %SL: body depth 33, head length 32–33. Measurements as %HL: snout length 45–50, orbit length 19–20, barbel length 77–91. Distal tip of the last ray of the second dorsal fin elongate, reaching the caudal‐fin base. In fresh coloration, the lateral surface of the body from the origin of the second dorsal fin forward to the head is reddish black; belly red; posterior body whitish yellow. Oblique yellow and blue streaks are present around the eye. Two white longitudinal stripes are present on the side of the body: the first stripe extends from the snout tip, passes above the eye, and reaches the origin of the second dorsal fin; the second stripe extends from the posterior margin of the lower jaw, passes below the eye, and reaches to below the fourth spine of the first dorsal fin. A black blotch is present on the lateral line, vertically aligned with the posterior end of the second dorsal fin. Barbels reddish brown. Scales on the dorsum and posterior body have conspicuous silvery‐white centers. Both dorsal fins reddish brown; second dorsal fin has yellow horizontal stripes. Pectoral and pelvic fins reddish brown. Anal fin pale dark red with seven yellow stripes. Caudal fin yellowish red, usually with a red blotch at the base; lower lobe with a broad black margin.

*Distribution*: Western Pacific: from the Moluccas and the Philippines to the Samoa Islands, north to the Ryukyu Islands and south to New Caledonia and Tonga; also recorded from Palau, the Caroline Islands, and the Marshall Islands in Micronesia. In China, the northern and southern South China Sea, and waters off Taiwan.

###### 3.1.1.2.4 Parupeneus indicus

Dorsal fins VIII, 9; anal fin I‐7; pectoral fin 15–16; pelvic fin I‐5; gill rakers 5–8 + 12–18 (Table 3). Size of specimens examined: SL 111.1–247.8 mm (*n* = 16). Measurements as %SL: body depth 29–33, head length 26–33. Measurements as %HL: snout length 33–48, orbit length 17–26, barbel length 63–91. Dorsum brown; lower body and belly white. Two parallel, irregular, narrow white lines are present below the eye, extending from the upper jaw, passing beneath the eye, to the posterior margin of the opercle. Distal tip of barbels are pale red; posterior half is white. Posterior margins of scales are black. In fresh coloration, a blue stripe extends from the snout tip, passing through the eye, and reaches the margin of the opercle; this stripe is inconspicuous in preserved specimens. An elongate oval golden‐yellow blotch is present on the side of the body below the interspace between the two dorsal fins. A large, round black blotch is present at the midlateral position of the caudal peduncle. First dorsal fin uniformly dark red; second dorsal fin with 3–4 brown horizontal bands. Pectoral fin and anal fin pale red; anal fin with four yellow oblique stripes. Caudal fin pale red; both upper and lower lobes with five rays bearing faint yellow horizontal stripes.

*Distribution*: Indo‐Pacific: Gulf of Aden and the Yemeni coast south of Oman, southward along the east coast of Africa to Port Alfred, South Africa; east to the Caroline and Samoan Islands; from southern Japan to southern Queensland. In China, the northern and southern South China Sea, the Beibu Gulf, and waters off Taiwan.

###### 3.1.1.2.5 Parupeneus barberinus

Dorsal fins VIII, 9; anal fin I‐7; pectoral fin 16–17; pelvic fin I‐5; gill rakers 4–7 + 16–21 (Table 3). Size of specimens examined: SL 268.2–343.3 mm (*n* = 5). Measurements as %SL: body depth 27–32, head length 29–36. Measurements as %HL: snout length 43–53, orbit length 12–20, barbel length 63–77. Body silvery white or pale red. Three blue stripes on the head extend across the eye to the margin of the opercle; scattered blue streaks are present on the upper part of the eye. Posterior margins of scales are reddish brown. A black longitudinal stripe is present on the side of the body, extending from the snout, passing through the eye, and reaching the posterior end of the second dorsal fin. A large, round black blotch is present at the midlateral position on the caudal peduncle. Barbels pale reddish brown. All fins reddish brown, with the first dorsal fin, pelvic fin, and caudal fin darker; pelvic and caudal fins with several yellow fin rays. Second dorsal fin and anal fin paler, each with 4–5 horizontal yellow stripes.

*Distribution*: Indo‐Pacific: Gulf of Aden and Gulf of Oman; southward along the east coast of Africa to Mossel Bay, South Africa; east to the Micronesian Islands, Line Islands, Marquesas Islands, and the Tuamotu Archipelago; from southern Japan to Australia and New Caledonia. In China: the northern and southern South China Sea, and waters off Taiwan.

###### 3.1.1.2.6 Parupeneus heptacantha

Dorsal fins VIII, 9; anal fin I‐7; pectoral fin 15–16; pelvic fin I‐5; gill rakers 5–7 + 16–22 (Table 3). Size of specimens examined: SL 129.6–182.0 mm (*n* = 5). Measurements as %SL: body depth 29–36, head length 28–32. Measurements as %HL: snout length 36–48, orbit length 19–28, barbel length 67–91. Dorsum red; lower body pale red; belly silvery white. Three irregular, narrow white lines are present on the head: the upper two extending through the eye to the margin of the opercle, and the lower one passing below the eye to the margin of the opercle. Barbels white. Posterior margins of scales are dark red. Three faint pale‐yellow longitudinal stripes are present on the upper side of the body; the first stripe lies immediately below the lateral line, with a red blotch located between the seventh and eighth spines of the first dorsal fin. First dorsal fin and pelvic fin pale red. Pectoral fin pale red, nearly transparent. Pelvic fin with faint yellow oblique streaks. Second dorsal fin and anal fin with alternating yellow and blue oblique bands. Caudal fin pale red, with several pale‐yellow soft rays.

*Distribution*: Indo‐West Pacific: Red Sea and the east coast of Africa to the Caroline Islands and Fiji. In China: the northern and southern South China Sea, the Beibu Gulf, and waters off Taiwan.

###### 3.1.1.2.7 Parupeneus ciliatus

Dorsal fins VIII, 9; anal fin I‐6; pectoral fin 14–15; pelvic fin I‐5; gill rakers 6–7 + 24–25 (Table 3). Size of specimens examined: SL 132.0–203.4 mm (*n* = 4). Measurements as %SL: body depth 34–36, head length 27–33. Measurements as %HL: snout length 37–42, orbit length 21–25, barbel length 59–67. In fresh coloration, body coloration varies. Adults with dorsum reddish brown and lower body paler; fins reddish yellow. Subadults with dorsum greenish brown and lower body paler, belly silvery white; fins yellowish green. Barbels yellow. Scale centers are conspicuously white, with red and greenish margins. Three dark longitudinal stripes are present on the side of the body: the first stripe originates on the head anterior to the eye, passes above the eye, and extends to the region of the second dorsal fin; the second stripe extends from the snout tip, passing through the eye, to the caudal‐fin base; the third stripe originates from the snout tip, passes below the eye, is conspicuous on the head but fades posteriorly, and merges with the second stripe. Three pale, narrow, parallel stripes are also present: The first lies above the first dark stripe and extends only to the posterior end of the second dorsal fin; the second stripe lies between the second and third dark stripes and terminates where the two dark stripes merge; the third stripe lies below the third dark stripe and is faint. A yellowish‐white saddle‐like blotch is present on the anterodorsal portion of the caudal peduncle, immediately followed posteriorly by a diffuse black saddle blotch; the black blotch is broader than the pale saddle and extends across the lateral line. Second dorsal fin and anal fin have scattered white spots.

*Distribution*: Indo‐Pacific: Western Indian Ocean eastward to the Marquesas Islands and the Tuamotu Archipelago; north to southern Japan, south to Australia and Rapa. In China, the northern and southern South China Sea, the Beibu Gulf, waters off Taiwan, and the East China Sea.

###### 3.1.1.2.8 Parupeneus biaculeatus

Dorsal fins VIII, 9; anal fin I‐7; pectoral fin 15–16; pelvic fin I‐5; gill rakers 4–7 + 18–23 (Table 3). Size of specimens examined: SL 114.5–217.0 mm (*n* = 32). Measurements as %SL: body depth 29–38, head length 28–34. Measurements as %HL: snout length 36–48, orbit length 19–28, barbel length 67–91. In fresh coloration, body coloration varies. Adults are reddish to reddish brown, dorsum darker, lower body paler, belly white; fins red and greenish. Subadults are pale greenish red, with dorsum darker, lower body paler, belly white. Barbels white. Three dark, parallel longitudinal stripes are present on the side of the body: the first stripe extends from the snout tip, passing through the eye, to the posterior end of the second dorsal fin; the second stripe extends from the snout tip, passing below the eye, to the caudal‐fin base. Three pale, narrow, parallel stripes are also present: The first stripe lies above the first dark stripe, slightly darker than the other two pale stripes and yellowish, extending only to the posterior end of the second dorsal fin; the second stripe lies between the two dark stripes, pale whitish, extending only to the posterior end of the second dorsal fin; the third stripe lies below the second dark stripe, pale whitish, extending to the caudal‐fin base. A yellowish‐white saddle‐like blotch is present on the anterodorsal portion of the caudal peduncle, immediately followed posteriorly by a diffuse black saddle blotch; the black blotch is broader than the pale saddle and extends across the lateral line.

*Distribution*: Western Pacific. In China, the northern South China Sea and waters off Taiwan.

###### 3.1.1.2.9 Parupeneus cyclostomus

Dorsal fins VIII, 9; anal fin I‐7; pectoral fin 14–15; pelvic fin I‐5; gill rakers 4–6 + 21–23 (Table 3). Size of specimens examined: SL 206.6–330.2 mm (*n* = 4). Measurements as %SL: body depth 26–31, head length 29–34. Measurements as %HL: snout length 40–53, orbit length 12–15, barbel length 77–111. In fresh coloration, two color forms were observed. In the first form, body grayish is yellow; scales bear blue spots; several irregular blue streaks are present below the eye; a yellow saddle‐like blotch is present on the upper part of the caudal peduncle; barbels are yellowish brown, grading distally; first dorsal fin grayish yellow, without spots or stripes; second dorsal fin and anal fin with alternating yellow and blue oblique bands; caudal fin grayish yellow with blue horizontal bands. In the second form, the head and body, both dorsal fins, pectoral fin, pelvic fin, anal fin, caudal fin, and barbels are all bright yellow; multiple irregular blue streaks are also present below the eye.

*Distribution*: Indo‐Pacific: Red Sea and the east coast of Africa, south to Durban, South Africa; east to the Hawaiian Islands, French Polynesia, and the Pitcairn Islands; north to the Ryukyu Islands and the Ogasawara Islands; south to Australia and southern Japan. In China, the southern South China Sea, the Beibu Gulf, waters off Taiwan, and the East China Sea.

###### 3.1.1.2.10 Parupeneus chrysopleuron

Dorsal fins VIII, 9; anal fin I‐7; pectoral fin 15; pelvic fin I‐5; gill rakers 4–7 + 17–21 (Table 3). Size of specimens examined: SL 113.3–177.0 mm (*n* = 24). Measurements as %SL: body depth 32–37, head length 25–31. Measurements as %HL: snout length 31–50, orbit length 22–32, barbel length 71–91. Body pale red, darker dorsally; lower body slightly paler anteriorly; belly silvery white. Oblique blue streaks are present around the eye. Barbels pale yellowish white. Posterior margins of scales are red. The upper side of the body bears a broad yellow longitudinal stripe extending from behind the eye to the caudal‐fin base. Dorsal fins red; second dorsal fin with two yellow stripes on the upper half, and the lower half uniformly red. Pectoral fin pale red, nearly transparent. Pelvic fin and anal fin pale red, with fin membranes nearly transparent, each with 2–3 yellow stripes; stripes on the pelvic fin less distinct than those on the anal fin. Caudal fin uniformly red.

*Distribution*: Indo‐West Pacific, discontinuously distributed from Japan (north to the coast of the Aomori Peninsula), Honshu, the Izu Peninsula, Kochi Prefecture, the Ogasawara Islands, and southeastern Taiwan (China); south to the Arafura Sea, the Northwest Shelf, and the Kimberley region of Western Australia; and to Geographe Bay. In China, the southern and northern South China Sea, the Beibu Gulf, waters off Taiwan, and the East China Sea.

##### 3.1.1.3 *Mulloidichthys* Whitley, 1929

Body elongated and slightly laterally compressed. Dorsal and ventral profiles relatively flat and similar. Head moderately large; snout blunt. Eye large, dorsolateral in position. Mouth small and subterminal; upper and lower jaws nearly equal in length. Vomer and palatines edentulous; both jaws with several rows of minute villiform teeth. A pair of barbels present on the underside of the chin. Two separate dorsal fins with similar height. Pectoral fin inserted posterior to the opercle; pelvic fin inserted below the pectoral‐fin base. Anal fin opposite and similar in shape to the second dorsal fin. Caudal fin deeply forked. Body covered with ctenoid scales. Lateral line complete, running parallel to the dorsal contour and extending to the caudal‐fin base.

###### 3.1.1.3.1 Mulloidichthys flavolineatus

Dorsal fins VI, 9; anal fin I‐6; pectoral fin 17; pelvic fin I‐5; gill rakers 7–8 + 17–20 (Table 2). Size of specimens examined: SL 138.6–213.7 mm (*n* = 5). Measurements as %SL: body depth 23–26, head length 28–31. Measurements as %HL: snout length 31–36, orbit length 25–29, barbel length 67–77. Second dorsal fin and anal fin with concave distal margins. Dorsum greenish brown; lower body and belly white. Barbels white. A yellow longitudinal stripe is present on the side of the body, extending from behind the eye to the caudal‐fin base, with a black blotch aligned with rays 5–7 of the second dorsal fin. Dorsal, pectoral, and caudal fins yellowish green; pelvic and anal fins white.

*Distribution*: Indo‐Pacific: Persian Gulf; Red Sea and eastern Africa to Hawaii, the Marquesas Islands, and the Ducie Islands; north to the Ryukyu Islands and the Ogasawara Islands, south to Lord Howe Island and the Rapa Islands. In China: the southern and northern South China Sea, the Beibu Gulf, and waters off Taiwan.

###### 3.1.1.3.2 Mulloidichthys vanicolensis

Dorsal fins VI, 9; anal fin I‐7; pectoral fin 17; pelvic fin I‐5; gill rakers 7–9 + 22–24 (Table 2). Size of specimens examined: SL 145.6–250.3 mm (*n* = 6). Measurements as %SL: body depth 27–29, head length 27–30. Measurements as %HL: snout length 28–36, orbit length 24–31, barbel length 67–91. Second dorsal fin and anal fin with concave distal margins. Dorsum reddish brown; lower body pale red; belly silvery white. Barbels white. A golden‐yellow longitudinal stripe present on the side of the body, extending from the snout tip, passing through the eye, to the caudal‐fin base, without blotches. All fins deep yellow, without spots or stripes.

*Distribution*: Indo‐Pacific: Red Sea, eastern Africa, the Comoro Islands, and the Mascarene Islands; east to the Hawaiian Islands and the Pitcairn Islands; north to southern Japan, south to Joseph Bonaparte Gulf, New South Wales, Lord Howe Island, Norfolk Island, the Kermadec Islands, New Caledonia, Tonga, and the Gambier Islands. In China, the southern and northern South China Sea, the Beibu Gulf, and waters off Taiwan.

Based on the voucher‐validated diagnostic characters summarized above, dichotomous identification keys to genera and species of Mullidae in the northern South China Sea are provided in Appendix 1.

#### 3.1.2 Morphometric Analysis

Morphometric data are presented in Tables 2 and 3. A total of 283 specimens were measured; 34 specimens with missing values in one or more morphometric variables were excluded, leaving 249 complete cases for multivariate analyses (*Upeneus* = 151, *Parupeneus* = 90, *Mulloidichthys* = 8). Based on the allometry‐adjusted multivariate morphometric matrix, PERMANOVA detected significant differences among the three genera (*F* = 27.246, *R* ^2^ = 0.181, *p* = 0.0001; Table 4), and multivariate dispersion differed among genera in the full dataset (betadisper, *p* = 0.0087). To address heterogeneous size ranges among genera, we conducted a sensitivity analysis restricted to specimens within the common standard‐length overlap across genera (SL = 138.6–198.5 mm; *n* = 76: *Parupeneus* = 51, *Upeneus* = 21, *Mulloidichthys* = 4). Within this size‐restricted subset, dispersion did not differ significantly among genera (betadisper, *p* = 0.1866), whereas genus‐level differences remained significant (*F* = 23.002, *R* ^2^ = 0.387, *p* = 0.0001), indicating that intergeneric differentiation persists under comparable body‐size conditions and is not driven solely by differences in size structure. A focused comparison between *Parupeneus* and *Mulloidichthys* also remained significant (*F* = 21.844, *R* ^2^ = 0.185, *p* = 0.0001; Table S3) with no evidence of unequal dispersion (betadisper, *p* = 0.3587); results were consistent in the two‐genus SL‐overlap subset (SL = 138.6–250.3 mm; *n* = 68: *Parupeneus* = 60, *Mulloidichthys* = 8; PERMANOVA: *F* = 18.479, *R* ^2^ = 0.219, *p* = 0.0001; betadisper: *p* = 0.4936), although Mulloidichthys was represented by a limited sample size. Taken together, PERMANOVA, dispersion tests, and size‐overlap sensitivity analyses support consistent multivariate morphological differentiation among genera after size adjustment, while substantial within‐genus variation remains.

**TABLE 4:** PERMANOVA results.

	df	*F*	*p*
(A) Main analysis (all specimens)			
Genus	2	27.246	0.0001
Residual	246		
Total	248		
			No. Perm = 9999
(B) Sensitivity analysis (common SL overlap, SL = 138.6–198.5 mm)			
Genus	2	23.002	0.0001
Residual	73		
Total	75		
			No. Perm = 9999

**FIGURE 2:**
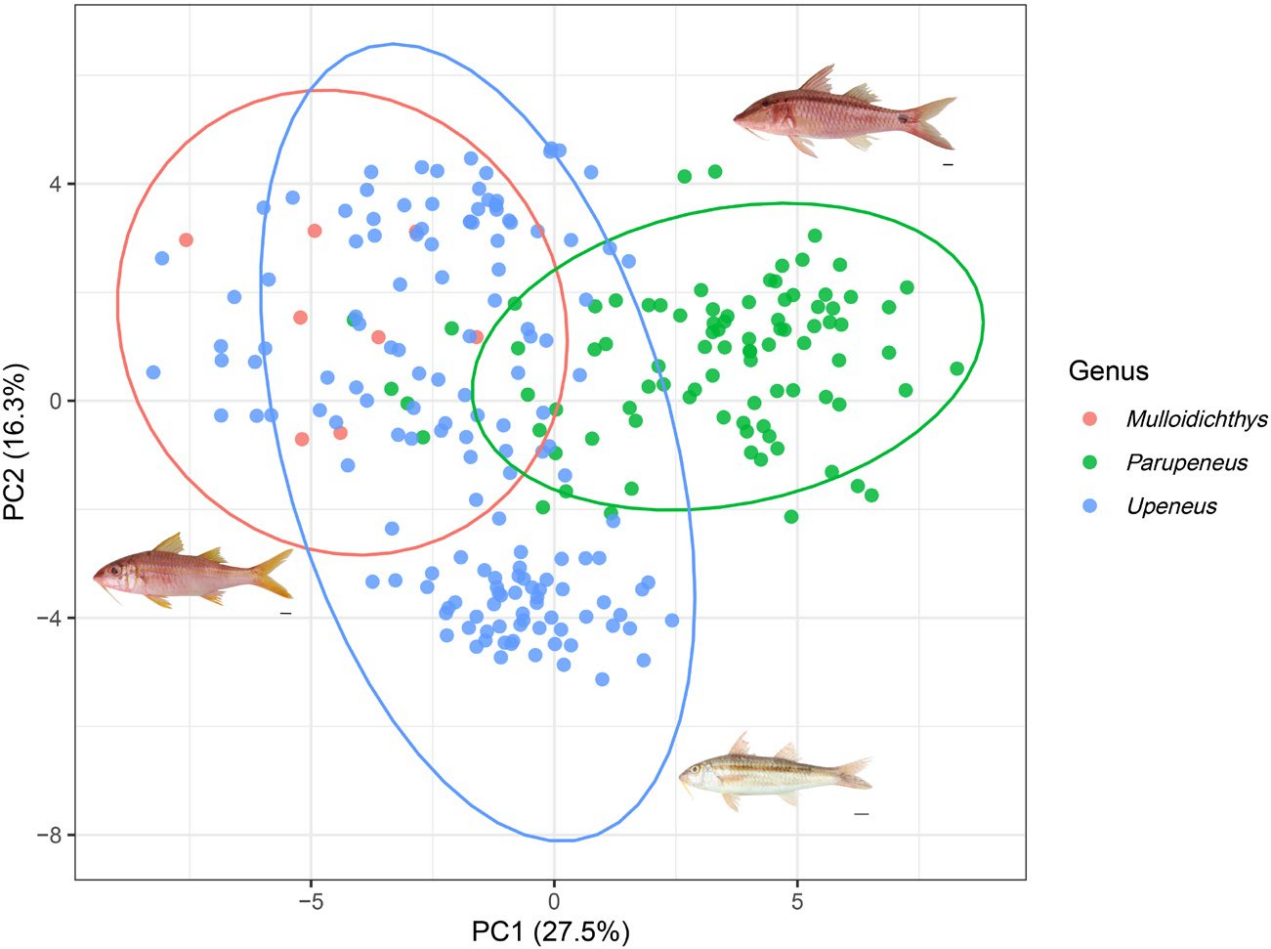
Principal component analysis of morphometric data for Mullidae. Representative species of the three genera are shown: *Mulloidichthys vanicolensis* , *Parupeneus barberinus* , and *Upeneus sundaicus* ; scale bars = 1 cm.

PCA of the allometry‐adjusted morphometric dataset showed that the first two principal components together explained 43.8% of the total variation (Figure 2). PC1 accounted for 27.5% of the variance and loaded most strongly (by absolute loadings) on depth‐related traits (e.g., head depth through eye, maximum head depth, body depth, body depth at the anal‐fin origin, and second dorsal‐fin depth), reflecting differences in vertical body profile. PC2 explained 16.3% of the variance and was associated mainly with caudal and head‐region metrics, including caudal‐peduncle length, second pre‐dorsal length, pectoral‐fin depth, caudal‐fin length, and snout length. In the PC1–PC2 morphospace, *Parupeneus* tended to separate from the other genera along PC1, whereas *Upeneus* and *Mulloidichthys* overlapped more extensively, with *Upeneus* showing a broader within‐genus spread.

### 3.2 Phylogenetic Relationships and Molecular Delimitation

NCBI BLASTn searches against the GenBank nucleotide database were used to cross‐check COI‐based taxonomic assignments. For most specimens, BLAST results were congruent with morphological identifications at the genus level. However, a small number of specimens showed either discordant species names relative to morphology or nonunique best hits at the species level (involving five species in total), and these cases occurred primarily within *Parupeneus* and *Upeneus*. For specimens with ambiguous BLAST outcomes, we additionally conducted cross‐validation using 16S rRNA. Notably, for specimens morphologically identified as *U. sundaicus* , COI BLAST results yielded high‐scoring matches to both *M. vanicolensis* and *U. sundaicus* , indicating genus‐level ambiguity, whereas the top 16S rRNA matches consistently corresponded to *Upeneus*, supporting the genus assignment inferred from morphology. In general, 16S rRNA provided consistent genus‐level corroboration but showed limited resolution among congeneric species; therefore, it was used only as auxiliary evidence. For specimens exhibiting discordant or ambiguous signals, final identifications were made by integrating voucher‐based morphological diagnoses with phylogenetic inference and species‐delimitation results.

#### 3.2.1 COI

**FIGURE 3:**
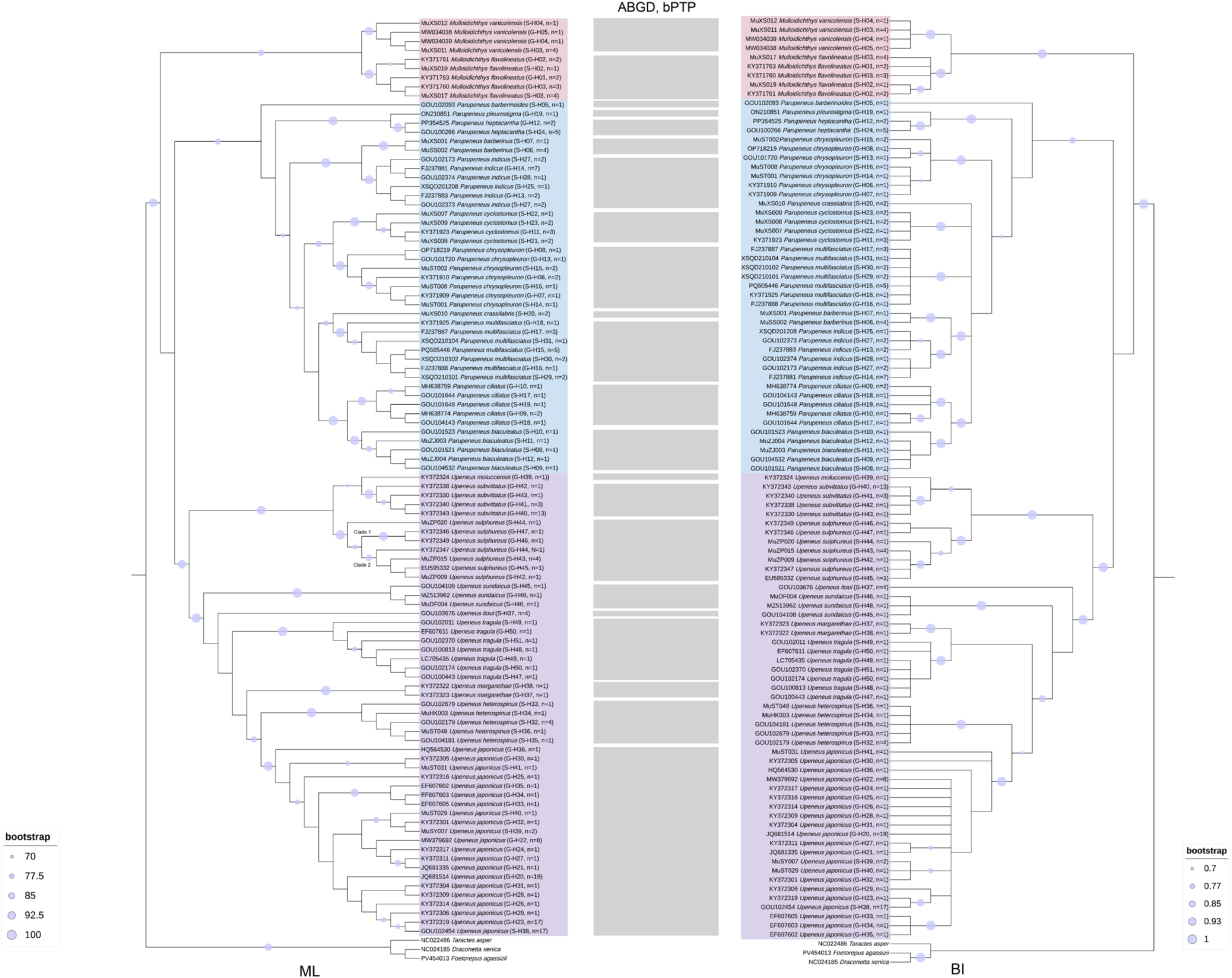
ML (left) and BI (right) phylogenies based on COI sequences. The central blocks depict the MOTU partitions inferred by ABGD and bPTP, where each continuous block corresponds to one MOTU.

An integrated dataset was constructed by combining 51 haplotypes generated in this study with 50 haplotypes retrieved from GenBank (representing three genera and 16 species) for subsequent analyses (Table S3–S5). The ML and BI analyses yielded identical overall topologies, recovering all genera and species as strongly supported monophyletic clades (Figure 3). The resulting phylogeny resolved goatfishes into two major lineages: one comprising all species of *Mulloidichthys* and the other including all remaining taxa. Within the latter lineage, two principal clades corresponding to *Parupeneus* and *Upeneus* were recovered. *Parupeneus* was further divided into two subclades, with *Parupeneus* 1 represented solely by *P. barberinoides* and *Parupeneus* 2 encompassing all other *Parupeneus* species. *Upeneus* was also split into two subclades: *Upeneus* 1 ( *U. moluccensis* , *U. subvittatus* , *U. sulphureus* ) and *Upeneus* 2 ( *U. sundaicus* , *U. itoui*, *U. tragula* , *U. margarethae*, *U. heterospinus*, *U. japonicus* ).

Based on COI sequences, intergeneric genetic distances were 17.21%–19.29% (mean 18.33% ± 1.05%, Table S6). The largest intergeneric distance was observed between *Parupeneus* and *Upeneus* (19.29%), whereas the smallest involved *Parupeneus* and *Mulloidichthys* (17.21%). Intrageneric genetic distances were 4.43%–12.46% (mean 9.39 ± 4.34%; Table S6), with the highest divergence in *Parupeneus* (12.46%) and the lowest in *Mulloidichthys* (4.43%). At the species level, the interspecific distance within *Mulloidichthys* was 7.78%. Within *Parupeneus*, interspecific distances were 5.14%–19.42% (mean 14.57% ± 3.46%; Table S7), with the maximum divergence between *P. barberinoides* and *P. chrysopleuron* (19.42%). Within *Upeneus*, interspecific distances were 7.93%–18.85% (mean 13.86% ± 3.64%; Table S7), with the largest distance between *U. sulphureus* and *U. sundaicus* (18.85%). Intraspecific genetic distances were low (0%–1.04%, mean 0.29% ± 0.24%; Table S8).

#### 3.2.2 16S rRNA

**FIGURE 4:**
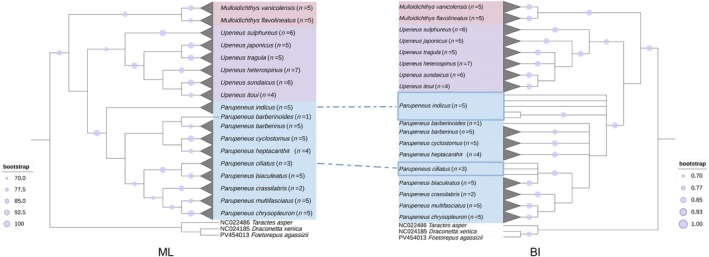
ML (left) and BI (right) phylogenies based on 16S rRNA sequences.

A total of 83 mitochondrial 16S rRNA sequences, representing 18 species from three genera, were obtained from goatfishes in the NSCS (Table S4). The ML tree resolved all examined species as monophyletic. In contrast, the BI tree showed *P. indicus* as unresolved within a polytomy, while all other species formed monophyletic groups (Figure 4). Overall, goatfishes were resolved into two strongly supported clades. Clade I comprised species of *Mulloidichthys*. Clade II included species of *Parupeneus* and *Upeneus*. Within *Upeneus*, two subclades were identified: the first subclade contained *U. sulphureus* , and the second comprised (i) *U. tragula* and *U. japonicus* , and (ii) *U. heterospinus*, *U. itoui*, and *U. sundaicus* .

Based on 16S rRNA sequences, intergeneric distances were 8.27%–11.00% (mean 9.62% ± 1.37%), with the maximum between *Parupeneus* and *Upeneus* (11.00%, Table S6). Intrageneric distances were 1.21%–4.76% (mean 3.44% ± 1.94%). At the species level, congeneric interspecific genetic distances in *Mulloidichthys* were 2.17%. Within *Parupeneus*, interspecific distances were 0.81%–12.02% (mean 5.22% ± 2.88%), with the maximum between *P. barberinoides* and *P. multifasciatus* (12.02%). Within *Upeneus*, interspecific distances were 1.40%–10.49% (mean 4.88% ± 3.49%), with the maximum between *U. sulphureus* and *U. tragula* (10.49%). Intraspecific distances were low (0%–0.15%, mean 0.04% ± 0.05%, Table S8).

#### 3.2.3 IRBP

**FIGURE 5:**
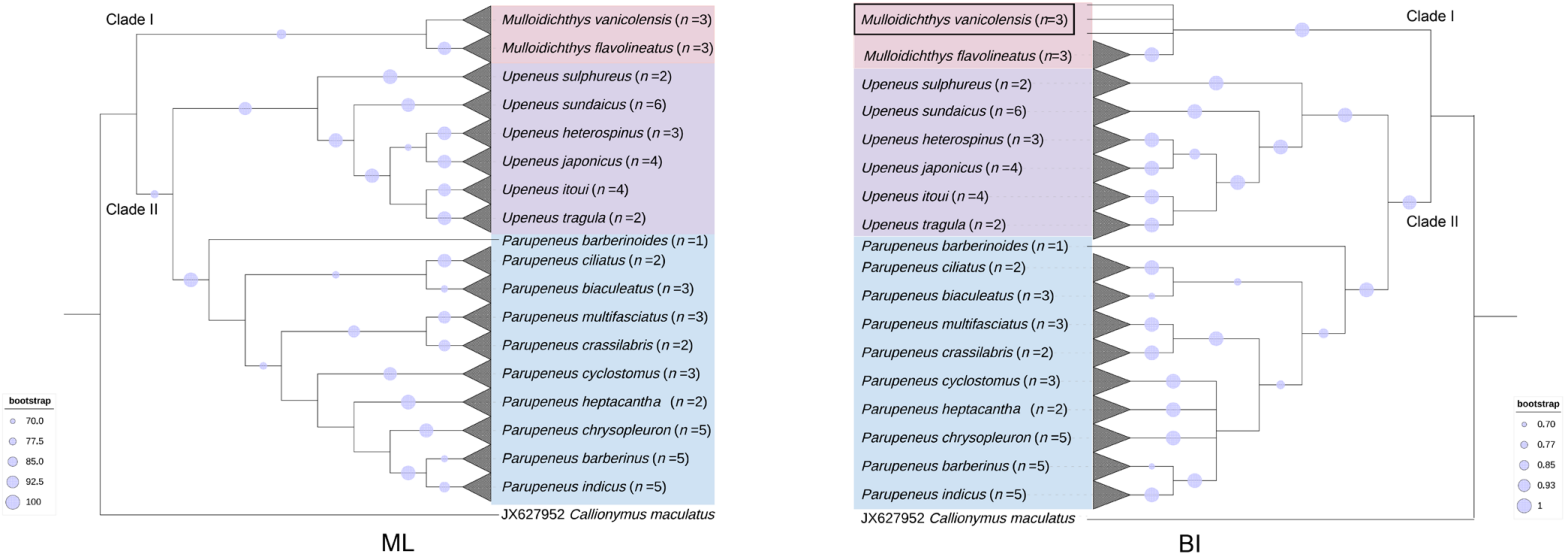
ML (left) and BI (right) phylogenies based on IRBP sequences.

A total of 58 IRBP sequences, representing 18 species from three genera, were obtained from goatfishes in the NSCS (Table S4). Phylogenetic analyses revealed that all species formed monophyletic clades in the ML tree. In contrast, the BI tree showed *M. vanicolensis* unresolved within a polytomy, while the remaining species were monophyletic (Figure 5). The topologies of the ML and BI trees were highly congruent, both recovering two major clades with high statistical support. Clade I comprised species of *Mulloidichthys*. Clade II included species of *Parupeneus* and *Upeneus*. Within *Upeneus*, two subclades were identified: the first subclade contained *U. sulphureus* ; the second subclade comprised two subgroups: (i) *U. sundaicus* and (ii) *U. tragula* , *U. itoui*, *U. heterospinus*, and *U. japonicus* .

IRBP‐based analyses demonstrated intergeneric distances of 10.27%–11.24% (mean 10.63% ± 0.54%), with the highest between *Parupeneus* and *Mulloidichthys* (11.24%). Intrageneric distances were 0.83%–3.19% (mean 1.92% ± 1.19%). At the species level, the congeneric interspecific distances in *Mulloidichthys* were 1.34%. Within *Parupeneus*, interspecific distances were 0.50%–2.91% (mean 2.05% ± 0.56%), with the maximum between *P. barberinoides* and *P. heptacantha* (2.91%). Within *Upeneus*, interspecific distances were 1.50%–7.88% (mean 4.19% ± 2.46%), with the maximum between *U. japonicus* and *U. sulphureus* (7.88%). Intraspecific distances were 0%–0.24% (mean 0.04% ± 0.08%, Table S8).

#### 3.2.4 COI + 16S rRNA

**FIGURE 6:**
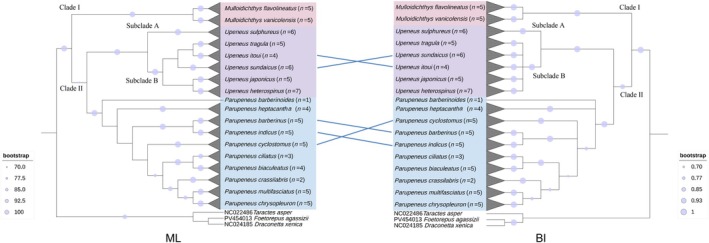
ML (left) and BI (right) phylogenies based on COI + 16S rRNA sequences.

Concatenated analysis of 83 COI and 16S rRNA sequences (3 genera, 18 species) yielded congruent ML and BI tree topologies, with all species receiving strong support for monophyly (Figure 6). Two primary clades were resolved within Mullidae: Clade I (*Mulloidichthys*) and Clade II (*Parupeneus* + *Upeneus*), consistent with topologies from 16S rRNA and IRBP analyses. Within *Upeneus*, two subclades were recovered: Subclade A ( *U. sulphureus* ) and Subclade B comprising (i) *U. sundaicus* , *U. tragula* , and *U. itoui*; (ii) *U. heterospinus* and *U. japonicus* .

Concatenated sequence analyses showed intergeneric distances of 12.29%–14.88% (mean 13.73%), with the highest between *Parupeneus* and *Upeneus* (14.88%, Table S6). At the species level, the congeneric interspecific distance in *Mulloidichthys* was 4.67% (between *M. flavolineatus* and *M. vanicolensis* ). Within *Parupeneus*, interspecific distances were 3.04%–14.80% (mean 9.70% ± 2.82%), with the maximum between *P. chrysopleuron* and *P. cyclostomus* (14.80%). Within *Upeneus*, interspecific distances were 5.46%–14.14% (mean 8.77% ± 3.56%), with the maximum between *U. sulphureus* and *U. sundaicus* (14.14%). Intraspecific distances were 0%–0.31% (mean 0.13 ± 0.10%, Table S8).

### 3.3 Species Delimitation

Using the trimmed and aligned COI dataset (101 sequences representing 22 nominal species across three genera), we conducted species delimitation with ABGD. To evaluate the effect of the relative gap width parameter (*X*) on delimitation outcomes, we performed a sensitivity analysis across *X* = 0.5, 0.8, 1.0, and 1.2 while keeping all other parameters unchanged. The results indicated that, across *X* = 0.5–1.0, ABGD consistently recovered 22 molecular operational taxonomic units (MOTUs) over a broad range of prior intraspecific divergence values (*p* = 0.002783–0.035938), and the initial and recursive partitions were identical across this stability plateau (Figure S1). Accordingly, we report the representative partition within this plateau (*X* = 1.0, Partition 6; *p* = 0.012915) as the primary ABGD delimitation result, with each nominal species corresponding to a single MOTU (Figure 3). Of these, 18 MOTUs correspond to sequences generated from our collected specimens, whereas the remaining four MOTUs are represented only by GenBank reference sequences.

bPTP delimitation, based on the unrooted ML tree inferred from the same COI dataset (using default settings) recovered 22 MOTUs under both the maximum‐likelihood estimate and the Bayesian consensus solution, fully congruent with the 22 nominal species (Figure 3). Likewise, 18 of these MOTUs correspond to our sampled specimens, and the remaining four are represented only by GenBank sequences.

## 4 Discussion

### 4.1 Morphological Taxonomy

Accurate taxonomy and reliable species identification provide a fundamental basis for biological research (Luo et al. 2018). In this study, we collected 18 goatfish species from the NSCS, belonging to three genera, *Upeneus* (6 species), *Parupeneus* (10 species), and *Mulloidichthys* (2 species). Phylogenetic analyses based on three markers (COI, 16S rRNA, and IRBP) recovered *Upeneus*, *Parupeneus*, and *Mulloidichthys* as well‐supported monophyletic genera within our dataset, consistent with morphology‐based generic assignments. Morphometric analyses further showed that the three genera were significantly differentiated overall based on allometry‐adjusted linear morphometric traits, as supported by PERMANOVA. This result remained significant in the sensitivity analysis restricted to the standard‐length overlap among genera, indicating that intergeneric differentiation persists under comparable body‐size conditions and is not driven solely by differences in size structure. However, the allometry‐adjusted linear morphometric dataset revealed partial overlap among the three genera in PC1–PC2 morphospace, indicating that intergeneric separation cannot be fully achieved using overall body‐proportion differences derived from linear measurements alone. Importantly, this overlap does not indicate an absence of morphological differentiation because PC1 and PC2 together explained only 43.8% of the total variation, and substantial within‐genus variation remained. This apparent discrepancy between molecularly supported generic boundaries and overlapping morphospace may reflect a combination of morphological convergence under similar ecological and functional contexts (Muschick et al. 2012), environmentally driven phenotypic plasticity (Olsson and Eklöv 2005), and ontogenetic variation among individuals (Fragkoulis et al. 2021; Spoljaric and Reimchen 2011). In addition, the relatively limited sample size of *Mulloidichthys* should be taken into account when interpreting its position in morphospace. Nevertheless, PC1 was dominated by depth‐related traits, including body depth and head depth, with *Parupeneus* clustering toward positive PC1 scores and exhibiting relatively greater body and head depth, whereas *Mulloidichthys* and *Upeneus* tended to occupy negative PC1 scores and overlapped more extensively. This trend is consistent with the primary body shape axis identified by Nash et al. (2022) using geometric morphometrics, in which *Parupeneus* is generally deeper‐bodied, whereas *Upeneus* and *Mulloidichthys* are relatively more slender. Overall, in Mullidae, body coloration and gross body shape alone often have limited diagnostic value. Reliable species‐level identification instead relies on systematic comparisons of meristic counts and proportional measurements, supplemented, when diagnostically informative, by relatively stable combinations of stripes and bands (Uiblein and Heemstra 2010; Uiblein et al. 2020, 2024; Uiblein 2021; Uiblein and Motomura 2021; Mittelheiser et al. 2022).

During taxonomic verification, we re‐examined several potentially confusable species with reference to recent taxonomic revisions of Mullidae, particularly Uiblein et al. (2024). Regarding *U. pori* , reported by Luo et al. (2023) as a new record from the South China Sea, our reassessment indicates that the voucher specimen should be identified as *U. itoui*. The specimen reported by Luo et al. (2023) has a gill‐raker count of 5–7 + 15–16 (total 21–23), which is inconsistent with *U. pori* . Uiblein et al. (2024) subdivided the *pori* species group into two subgroups, with a total gill‐raker count of 21–25 in subgroup I (including *U. itoui*) and 25–29 in subgroup II (including *U. pori* ). Accordingly, the gill‐raker count of the specimen agrees with subgroup I rather than subgroup II. In addition, based on multiple morphometric traits, including pelvic‐fin length, pectoral‐fin length, and first dorsal‐fin height, the specimen matches the diagnosis of *U. itoui* (Table 1). Biogeographic evidence further supports this reassignment. *Upeneus pori* is currently known from the western Indian Ocean, whereas *U. itoui* is primarily distributed in the western Pacific, with relatively high abundance in Japan and only rare records from southern Taiwan. Therefore, the voucher specimen collected by Luo et al. (2023) from the northwestern coastal South China Sea off China represents an additional distributional record of *U. itoui* for this region, thereby updating and refining its documented geographic range.

In addition, we systematically compared the morphological characters of the specimens examined in this study and cross‐checked them against Chinese and international taxonomic revisions, field guides, and identification keys for Mullidae. We found that the descriptions and illustrations attributed to *P. trifasciatus* in *Fishes of the South China Sea* (Institute of Zoology 1962), *Systematic Synopsis of Chinese Fishes* (Cheng and Zheng 1987), *Fishes of Taiwan* (Shen 1993), and *Marine Fishes of China* (Chen and Zhang 2015) more closely match the diagnostic characters of *P. multifasciatus* , particularly with respect to the number of lateral bars and their extent. Specifically, the depicted and described individuals show five dark lateral bars that extend posteriorly only to mid‐body, with only the posterior three bars being distinct, which is characteristic of *P. multifasciatus* . In contrast, *P. trifasciatus* typically exhibits two prominent dark lateral bars that extend ventrally to the belly. This confusion likely traces back to historical name usage. Günther (1874) applied the name *P. multifasciatus* to the taxon that is currently recognized as *P. trifasciatus* , which may have contributed to subsequent misapplications of the scientific name in classic ichthyological references. Moreover, *Marine Fishes of China* (Chen and Zhang 2015) treated *P. multifasciatus* as a junior synonym of *P. trifasciatus* , which is inconsistent with recent taxonomic revisions; current evidence supports treating them as two valid species. Based on the above morphological comparisons and literature verification, we consider that records of *P. trifasciatus* in these works are more appropriately reassigned to *P. multifasciatus* .

Within *Parupeneus*, *P. biaculeatus* and *P. ciliatus* are morphologically similar, and their body coloration changes during ontogeny. In both species, juveniles are brownish green, subadults are reddish brown, and adults are predominantly red. Both species bear two dark longitudinal stripes extending from the snout across the eye, and a paired pale and dark saddle mark on the dorsal side of the caudal peduncle. The key diagnostic characters separating the two species are that *P. biaculeatus* has white barbels and typically lacks distinct white spots on the anal fin and second dorsal fin, whereas *P. ciliatus* has yellow barbels and commonly bears scattered white spots on the anal and second dorsal fins. Notably, in *Fishes of the South China Sea* (Institute of Zoology 1962) and *Systematic Synopsis of Chinese Fishes* (Cheng and Zheng 1987), *P. biaculeatus* was treated as a junior synonym of *P. ciliatus* . However, the descriptions and illustrations in these works do not highlight the key distinguishing characters, particularly the white spots on the second dorsal and anal fins, and the characters depicted are more consistent with *P. biaculeatus* . Given that both species have been recorded from the South China Sea, such incomplete accounts that fail to emphasize diagnostic traits may increase the risk of misidentification in subsequent taxonomic and survey studies. Therefore, in future identifications and data synthesis, we recommend prioritizing the diagnostic character combinations emphasized in recent taxonomic revisions and, whenever possible, verifying identifications using voucher specimens and photographic documentation.

### 4.2 Molecular Evidence and Species Delimitation

The choice of molecular markers is critical for species identification and delimitation. In this study, we conducted single‐locus phylogenetic analyses of COI, 16S rRNA, and IRBP, and further inferred phylogenetic relationships based on the concatenated COI + 16S rRNA dataset, with the aim of providing molecular support for species identification and delimitation of specimens collected from the NSCS. The results recovered *Mulloidichthys*, *Upeneus*, and *Parupeneus* as three well‐supported monophyletic genera within our dataset. Under our sampling scheme and marker combination, *Mulloidichthys* formed a distinct clade, whereas *Upeneus* and *Parupeneus* were recovered as sister taxa. This intergeneric topology differs from frameworks based on ultraconserved elements (Nash et al. 2022) and mitochondrial genomes (Luo et al. 2024), and may reflect differences in phylogenetic signal for resolving deeper intergeneric nodes across marker types and locus sampling (Wallis et al. 2017; Alda et al. 2021). Within *Upeneus*, two clades were resolved: one comprising only *U. moluccensis* , *U. subvittatus* and *U. sulphureus* , and the other including *U. sundaicus* , *U. itoui*, *U. tragula* , *U. margarethae*, *U. heterospinus*, and *U. japonicus* . Notably, previous studies based on ultraconserved elements (Nash et al. 2022), mitochondrial genomes (Luo et al. 2024), and morphology (Kim 2002) have likewise supported a division of *Upeneus* into two major clades. Within *Parupeneus*, different markers yielded inconsistent placements for early‐diverging nodes. Phylogenies based on IRBP and the concatenated dataset supported *P. barberinoides* as the earliest‐diverging lineage within *Parupeneus*, consistent with Nash et al. (2022). In contrast, the 16S rRNA phylogeny placed *P. indicus* as the earliest‐diverging lineage, whereas mitochondrial‐genome phylogenies placed *P. heptacantha* in that position. Such topological discordance may reflect limited phylogenetic signal at individual loci, incomplete lineage sorting (Toews and Brelsford 2012), and heterogeneity in substitution rates and patterns among molecular markers (Betancur‐R et al. 2013). In addition, in single‐locus BI analyses, some species (e.g., *P. indicus* and *P. ciliatus* ) were not consistently recovered as monophyletic, further suggesting that single loci may provide insufficient information for resolving these nodes. By contrast, the concatenated COI + 16S rRNA dataset substantially increased species‐level resolution, recovered monophyly for all species, and yielded higher support values. This result indicates that adding loci can improve the stability and support of species‐level topologies (Betancur‐R et al. 2013; Al‐Amry et al. 2024), thereby strengthening congruence with morphology‐based identifications and species‐delimitation results.

The two molecular species‐delimitation approaches employed in this study (ABGD and bPTP) produced concordant results, recovering 18 MOTUs among our sampled specimens, with each morphologically identified species corresponding to a unique MOTU. This pattern is consistent with voucher‐based morphological identifications and with the species‐level monophyly recovered in both the single‐locus ML trees and the concatenated‐gene phylogenies, collectively supporting the robustness of the species delimitations in this study across multiple analytical frameworks.

Phylogenetic analysis integrating COI sequences from this study with public GenBank records resolved two well‐supported intraspecific clades within *U. sulphureus* (Clade 1: BS = 96.9; Clade 2: BS = 85.4). Clade 1 comprised GenBank sequences from the Beibu Gulf, whereas Clade 2 included our samples from Zhanjiang (Guangdong) together with several GenBank sequences with broad locality annotations referring to the South China Sea off China. Notably, the Beibu Gulf and the coastal waters off Zhanjiang lie on the western and eastern sides of the Leizhou Peninsula, respectively, and are connected via the Qiongzhou Strait. Overall, the clade partition shows a weak geographic tendency and may therefore provide preliminary evidence for geographically structured COI variation within this species. Recent taxonomic work on the *U. sulphureus* species group further suggests that this lineage may exhibit complex intraspecific variation; for example, barbel length shows a bimodal distribution at a broad geographic scale, yet this difference is not concordant with other morphometric traits, meristic counts, or coloration and pattern characters (Uiblein et al. 2025). However, haplotype analyses further showed that haplotype distributions were not uniquely associated with the phylogenetic clades (Table S9). This incomplete coupling between phylogenetic clustering and haplotype patterns may reflect the relatively recent divergence of the two clades and the limited information content of a single mitochondrial locus (Zhou et al. 2021; Wu et al. 2024). In addition, the coarse or imprecise locality metadata associated with some public sequences may have partly obscured associations between lineages and specific geographic sources. To further evaluate whether these clades represent reproducible intraspecific lineage divergence, as opposed to an apparent pattern driven by the limitations of a single‐marker dataset and uncertainty in sample metadata, future work should increase sampling density in key regions, such as around the Qiongzhou Strait, and incorporate multilocus nuclear markers for comprehensive validation. At present, these results are most conservatively viewed as a weak geographic signal in COI variation within *U. sulphureus* in the northern South China Sea, rather than evidence for cryptic species or distinct lineages, and should be re‐evaluated with denser sampling and multilocus nuclear data.

In light of the node‐level instability observed at several nodes in our single‐locus analyses, challenges remain in applying molecular data to species delimitation. Speciation is a continuous and dynamic process, and during transitional stages in which phenotypic differentiation and reproductive isolation have not yet become fully stabilized, different species concepts and delimitation criteria may yield different inferences, leading to disagreement over species boundaries (De Queiroz 2007; Zhou et al. 2021). Although integrative approaches that combine molecular evidence, such as DNA sequences, with morphology and other data have been widely applied to species delimitation (Puillandre et al. 2012), their effectiveness depends strongly on the completeness of the underlying data. For taxa that remain poorly studied or are represented by incomplete data, taxonomic resolution may remain limited (Kekkonen and Hebert 2014). Moreover, bridging the gap from species delimitation to formal species description remains a practical challenge in taxonomy. Even when certain lineages have accumulated sufficient evidence to support their independence, the absence of systematic morphological descriptions and traceable voucher documentation can constrain their usability and cumulative value in subsequent research (Pante et al. 2015).

## 5 Conclusions

This study integrated morphological evidence with multilocus molecular markers, including mitochondrial COI and 16S rRNA genes and the nuclear gene IRBP, to identify and delimit goatfish species from the NSCS, confirming a total of 18 species belonging to three genera. Phylogenetic results indicate that, relative to single‐locus analyses, the concatenated dataset provides more stable species‐level monophyly and higher support values, thereby increasing the reliability of species delimitation. Comparative analyses incorporating COI sequences from public databases suggest that some species may exhibit weak geographic signals of intraspecific genetic heterogeneity; however, their taxonomic implications require further evaluation through denser sampling and multilocus nuclear data. Based on meristic characters and external body patterning of voucher specimens, we constructed an identification key to the 18 goatfish species recognized in this study to facilitate future surveys, monitoring, and specimen identification.

## Author Contributions

**Xiafang Li:** data curation (lead), formal analysis (lead), investigation (equal), methodology (equal), software (lead), writing – original draft (lead), writing – review and editing (lead). **Zhisen Luo:** data curation (equal), investigation (equal), methodology (equal), writing – review and editing (equal). **Murong Yi:** formal analysis (equal), investigation (equal). **Bin Kang:** conceptualization (equal), methodology (equal). **Xiongbo He:** formal analysis (equal), investigation (equal). **Liangliang Huang:** writing – review and editing (lead). **Hung‐Du Lin:** methodology (equal), writing – review and editing (equal). **Yunrong Yan:** conceptualization (equal), funding acquisition (equal), methodology (equal), project administration (lead), writing – review and editing (lead).

## Funding

This work was supported by the National Natural Science Foundation of China (42406098, U20A2087) and the Department of Science and Technology of Guangdong Province (2024B1212040002).

## Conflicts of Interest

The authors declare no conflicts of interest.

## Supporting information


**Figure S1:** Sensitivity of ABGD delimitations to the relative gap width (X).
**Table S1:** Primers used for amplifying partial sequences of COI, 16S rRNA, and IRBP genes.
**Table S2:** GenBank accession numbers for outgroup taxa.
**Table S3:** COI sequences from GenBank.
**Table S4:** List of species used in this study with museum numbers and GenBank accession numbers of COI, 16S rRNA, IRBP sequences.
**Table S5:** COI haplotype summary for this study (new sequences only).
**Table S6:** Intergeneric and intrageneric genetic distances (%) for Mullidae based on COI(this study+GenBank),16S rRNA(this study), IRBP(this study), and COI + 16S rRNA(this study) sequences.
**Table S7:** Intraspecific and interspecific genetic distances (%) based on COI (this study+GenBank) sequences.
**Table S8:** Intraspecific and interspecific genetic distances (%) based on 16S rRNA, IRBP, and COI + 16S rRNA sequences in this study.
**Table S9:** Sequence identity check and unified haplotype collapsing for six *Upeneus sulphureus* COI haplotypes grouped in the phylogeny.

## Data Availability

Data to support this study are available in the DRYAD (https://datadryad.org/dataset/doi:10.5061/dryad.5x69p8dhd) and GenBank at the National Center for Biotechnology Information (accession numbers: COI: PV834040–PV834124, 16S rRNA: PV833940–PV834022, IRBP: PV839439–PV839496). Sequences were accessed on 25–26 June 2025.
