# Supplementary material for: Integrative Taxonomic Revision of Goatfishes (Mullidae) in the Northern South China Sea Based on Morphology and Multilocus Molecular Data
Source: Ecol Evol. 2026 Apr 10;16(4):e73378. doi: 10.1002/ece3.73378 (PMC13068499; doi:10.1002/ece3.73378)
Supplement: Supplementary file 1 — Figure S1: Sensitivity of ABGD delimitations to the relative gap width (X). Table S1: Primers used for amplifying partial sequences of COI, 16S rRNA, and IRBP genes. Table S2: GenBank accession numbers for outgroup taxa. Table S3: COI sequences from GenBank. Table S4: List of species used in this study with museum numbers and GenBank accession numbers of COI, 16S rRNA, IRBP sequences. Table S5: COI haplotype summary for this study (new sequences only). Table S6: Intergeneric and intrageneric genetic distances (%) for Mullidae based on COI(this study+GenBank),16S rRNA(this study), IRBP(this study), and COI + 16S rRNA(this study) sequences. Table S7: Intraspecific and interspecific genetic distances (%) based on COI (this study+GenBank) sequences. Table S8: Intraspecific and interspecific genetic distances (%) based on 16S rRNA, IRBP, and COI + 16S rRNA sequences in this study. Table S9: Sequence identity check and unified haplotype collapsing for six Upeneus sulphureus COI haplotypes grouped in the phylogeny. [file ECE3-16-e73378-s001.docx]

****Supplementary Figure****

****
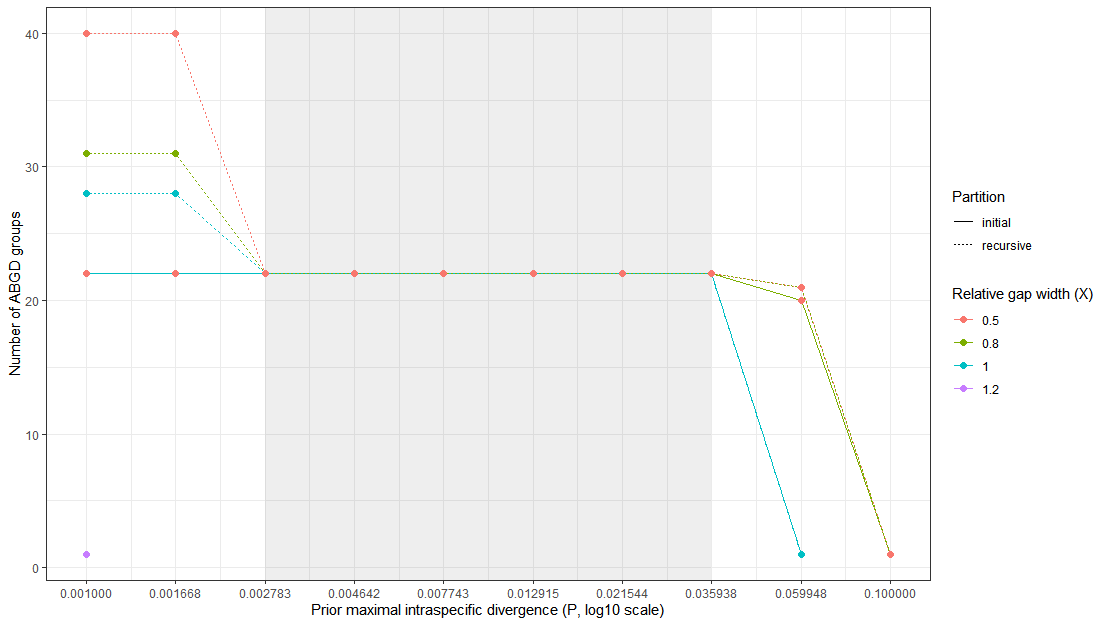
****

****Figure S1 Sensitivity of ABGD delimitations to the relative gap width (X).****

****Supplementary Table****

****Table S1**** Primers used for amplifying partial sequences of *COI*, 16S rRNA, and IRBP genes.

| **Genes** | **Primer name and sequence** | **Type** | Reference |
| --- | --- | --- | --- |
| *COI* | FishF1：5'-TCAACCAACCACAAAGACATTGGCAC-3' | Forward primer | (Ward et al. 2005) |
|  | FishR1：5'-TAGACTTCTGG GTGGCCAAACAATCA-3' | Reverse primer |  |
| 16S rRNA | 16Sbr-F：5'-CGCCTGTTTATCAAAAACAT-3' | Forward primer | (López et al. 2000) |
|  | 16Sbr-R：5'-CCGGTCTGAACTCAGATCACGT-3' | Reverse primer |  |
| IRBP | IRBP U110：5'-TGGACAAYT ACTGCTCRCCAGA-3' | Forward primer | (Dettaï and Lecointre 2008) |
|  | IRBP L936：5'-CACGGAGGYTGAYNATCTTGAT-3' | Reverse primer |  |

****Table S2**** GenBank accession numbers for outgroup taxa.

| **Species** | **GenBank accession numbers** | | |
| --- | --- | --- | --- |
|  | **COI** | **16S rRNA** | **IRBP** |
| *Draconetta xenica* | NC024185 | NC024185 | — |
| *Foetorepus agassizii* | PV454013 | PV454013 | — |
| *Taractes asper* | NC022486 | NC022486 | — |
| *Callionymus maculatus* | — | — | JX627952 |

Note: — not used in phylogenetic analyses.

****Table S3** COI sequences from GenBank.**

****Table S3 A** Haplotype summary (n = 50).**

| ****Hap ID**** | ****Species**** | ****Number**** | ****Representative GenBank** accession** |
| --- | --- | --- | --- |
| G-H01 | *M. flavolineatus* | 2 | KY371763 |
| G-H02 | *M. flavolineatus* | 2 | KY371761 |
| G-H03 | *M. flavolineatus* | 3 | KY371760 |
| G-H04 | *M. vanicolensis* | 1 | MW034039 |
| G-H05 | *M. vanicolensis* | 1 | MW034038 |
| G-H06 | *P. chrysopleuron* | 2 | KY371910 |
| G-H07 | *P. chrysopleuron* | 1 | KY371909 |
| G-H08 | *P. chrysopleuron* | 1 | OP718219 |
| G-H09 | *P. ciliatus* | 2 | MH638774 |
| G-H10 | *P. ciliatus* | 1 | MH638759 |
| G-H11 | *P. cyclostomus* | 3 | KY371923 |
| G-H12 | *P. heptacantha* | 2 | PP354525 |
| G-H13 | *P. indicus* | 2 | FJ237883 |
| G-H14 | *P. indicus* | 7 | FJ237881 |
| G-H15 | *P. multifasciatus* | 5 | PQ505446 |
| G-H16 | *P. multifasciatus* | 1 | FJ237888 |
| G-H17 | *P. multifasciatus* | 3 | FJ237887 |
| G-H18 | *P. multifasciatus* | 1 | KY371925 |
| G-H19 | *P. pleurostigma* | 1 | ON210851 |
| G-H20 | *U. japonicus* | 19 | JQ681514 |
| G-H21 | *U. japonicus* | 1 | JQ681335 |
| G-H22 | *U. japonicus* | 8 | MW379692 |
| G-H23 | *U. japonicus* | 17 | KY372319 |
| G-H24 | *U. japonicus* | 1 | KY372317 |
| G-H25 | *U. japonicus* | 1 | KY372316 |
| G-H26 | *U. japonicus* | 1 | KY372314 |
| G-H27 | *U. japonicus* | 1 | KY372311 |
| G-H28 | *U. japonicus* | 1 | KY372309 |
| G-H29 | *U. japonicus* | 1 | KY372306 |
| G-H30 | *U. japonicus* | 1 | KY372305 |
| G-H31 | *U. japonicus* | 1 | KY372304 |
| G-H32 | *U. japonicus* | 1 | KY372301 |
| G-H33 | *U. japonicus* | 1 | EF607605 |
| G-H34 | *U. japonicus* | 1 | EF607603 |
| G-H35 | *U. japonicus* | 1 | EF607602 |
| G-H36 | *U. japonicus* | 1 | HQ564530 |
| G-H37 | *U. margarethae* | 1 | KY372323 |
| G-H38 | *U. margarethae* | 1 | KY372322 |
| G-H39 | *U. moluccensis* | 1 | KY372324 |
| G-H40 | *U. subvittatus* | 13 | KY372343 |

****Table S3 A** continued**

| ****Hap ID**** | ****Species**** | ****Number**** | ****Representative GenBank** accession** |
| --- | --- | --- | --- |
| G-H41 | *U. subvittatus* | 3 | KY372340 |
| G-H42 | *U. subvittatus* | 1 | KY372338 |
| G-H43 | *U. subvittatus* | 1 | KY372330 |
| G-H44 | *U. sulphureus* | 1 | KY372347 |
| G-H45 | *U. sulphureus* | 1 | EU595332 |
| G-H46 | *U. sulphureus* | 1 | KY372349 |
| G-H47 | *U. sulphureus* | 1 | KY372346 |
| G-H48 | *U. sundaicus* | 1 | MZ513962 |
| G-H49 | *U. tragula* | 6 | LC705435 |
| G-H50 | *U. tragula* | 1 | EF607611 |

****Table S3 B** Accession-to-haplotype mapping (n = 132).**

| **Species** | **Hap ID** | **GenBank accession** | **Sampling locality** |
| --- | --- | --- | --- |
| *Mu. flavolineatus* | G-H01 | KY371763 | Nansha Islands, China |
| *Mu. flavolineatus* | G-H01 | KY371762 | Nansha Islands, China |
| *Mu. flavolineatus* | G-H02 | KY371761 | Nansha Islands, China |
| *Mu. flavolineatus* | G-H02 | KY371758 | Nansha Islands, China |
| *Mu. flavolineatus* | G-H03 | KY371760 | Nansha Islands, China |
| *Mu. flavolineatus* | G-H03 | KY371759 | Nansha Islands, China |
| *Mu. flavolineatus* | G-H03 | KY371757 | Nansha Islands, China |
| *Mu. vanicolensis* | G-H04 | MW034039 | Nansha Islands, China |
| *Mu. vanicolensis* | G-H05 | MW034038 | Nansha Islands, China |
| *P. chrysopleuron* | G-H06 | KY371910 | Northern South China Sea, China |
| *P. chrysopleuron* | G-H07 | KY371909 | Qiongzhou Strait, China |
| *P. chrysopleuron* | G-H08 | OP718219 | Zhanjiang, Guangdong, China |
| *P. ciliatus* | G-H09 | MH638774 | Dongsha Islands, China |
| *P. ciliatus* | G-H09 | MH638760 | Dongsha Islands, China |
| *P. ciliatus* | G-H10 | MH638759 | Dongsha Islands, China |
| *P. cyclostomus* | G-H11 | KY371923 | Nansha Islands, China |
| *P. cyclostomus* | G-H11 | OP718223 | China |
| *P. cyclostomus* | G-H11 | MW034196 | Nansha Islands, China |
| *P. heptacantha* | G-H12 | PP354525 | South China Sea, China |
| *P. heptacantha* | G-H12 | KY371924 | Beibu Gulf, China |
| *P. indicus* | G-H13 | OL581685 | Zhanjiang, Guangdong, China |
| *P. indicus* | G-H13 | FJ237883 | South China Sea, China |
| *P. indicus* | G-H13 | FJ237882 | South China Sea, China |
| *P. indicus* | G-H13 | FJ237880 | South China Sea, China |
| *P. indicus* | G-H13 | FJ237879 | South China Sea, China |
| *P. indicus* | G-H13 | FJ237878 | South China Sea, China |
| *P. indicus* | G-H13 | FJ237877 | South China Sea, China |
| *P. indicus* | G-H13 | MW034081 | Nansha Islands, China |
| *P. indicus* | G-H14 | FJ237881 | South China Sea, China |
| *P. indicus* | G-H14 | FJ237876 | South China Sea, China |
| *P. indicus* | G-H14 | FJ237875 | South China Sea, China |
| *P. indicus* | G-H14 | FJ237874 | South China Sea, China |
| *P. indicus* | G-H14 | FJ237873 | South China Sea, China |
| *P. indicus* | G-H14 | FJ237872 | South China Sea, China |
| *P. indicus* | G-H14 | FJ237871 | South China Sea, China |
| *P. multifasciatus* | G-H15 | PQ505446 | Wenchang, Hainan, China |
| *P. multifasciatus* | G-H15 | MW379591 | Tokin gulf, South China Sea, China |
| *P. multifasciatus* | G-H15 | KY371927 | Nansha Islands, China |
| *P. multifasciatus* | G-H15 | KY371926 | Nansha Islands, China |
| *P. multifasciatus* | G-H15 | OP718221 | China |
| *P. multifasciatus* | G-H16 | FJ237888 | South China Sea, China |

****Table S3 B** continued**

| **Species** | **Hap ID** | **GenBank accession** | **Sampling locality** |
| --- | --- | --- | --- |
| *P. multifasciatus* | G-H16 | FJ237886 | South China Sea, China |
| *P. multifasciatus* | G-H16 | FJ237885 | South China Sea, China |
| *P. multifasciatus* | G-H17 | FJ237887 | South China Sea, China |
| *P. multifasciatus* | G-H17 | FJ237884 | South China Sea, China |
| *P. multifasciatus* | G-H18 | KY371925 | Nansha Islands, China |
| *P. pleurostigma* | G-H19 | ON210851 | China |
| *U. japonicus* | G-H20 | JQ681514 | South China Sea, China |
| *U. japonicus* | G-H20 | MW379801 | Tokin gulf, South China Sea, China |
| *U. japonicus* | G-H20 | MW379687 | Tokin gulf, South China Sea, China |
| *U. japonicus* | G-H20 | MW379531 | Tokin gulf, South China Sea, China |
| *U. japonicus* | G-H20 | KY372321 | Northern South China Sea, China |
| *U. japonicus* | G-H20 | KY372320 | Northern South China Sea, China |
| *U. japonicus* | G-H20 | KY372307 | Northern South China Sea, China |
| *U. japonicus* | G-H20 | KY372302 | Northern South China Sea, China |
| *U. japonicus* | G-H20 | KY372318 | Nansha Islands, China |
| *U. japonicus* | G-H20 | KY372315 | Beibu Gulf, China |
| *U. japonicus* | G-H20 | KY372313 | Beibu Gulf, China |
| *U. japonicus* | G-H20 | KY372312 | Nansha Islands, China |
| *U. japonicus* | G-H20 | KY372310 | Nansha Islands, China |
| *U. japonicus* | G-H20 | KY372308 | Beibu Gulf, China |
| *U. japonicus* | G-H20 | KY372303 | Beibu Gulf, China |
| *U. japonicus* | G-H20 | KY372300 | Nansha Islands, China |
| *U. japonicus* | G-H20 | EF607606 | China |
| *U. japonicus* | G-H20 | EF607604 | China |
| *U. japonicus* | G-H20 | MN893179 | China |
| *U. japonicus* | G-H21 | JQ681335 | South China Sea, China |
| *U. japonicus* | G-H22 | MW379692 | Tokin gulf, South China Sea, China |
| *U. japonicus* | G-H23 | KY372319 | Northern South China Sea, China |
| *U. japonicus* | G-H23 | KY372299 | South China Sea, China |
| *U. japonicus* | G-H24 | KY372317 | Nansha Islands, China |
| *U. japonicus* | G-H25 | KY372316 | Nansha Islands, China |
| *U. japonicus* | G-H26 | KY372314 | Nansha Islands, China |
| *U. japonicus* | G-H27 | KY372311 | Nansha Islands, China |
| *U. japonicus* | G-H28 | KY372309 | Beibu Gulf, China |
| *U. japonicus* | G-H29 | KY372306 | Nansha Islands, China |
| *U. japonicus* | G-H30 | KY372305 | Nansha Islands, China |
| *U. japonicus* | G-H31 | KY372304 | Beibu Gulf, China |
| *U. japonicus* | G-H32 | KY372301 | Nansha Islands, China |
| *U. japonicus* | G-H33 | EF607605 | China |
| *U. japonicus* | G-H34 | EF607603 | China |
| *U. japonicus* | G-H35 | EF607602 | China |

****Table S3 B** continued**

| **Species** | **Hap ID** | **GenBank accession** | **Sampling locality** |
| --- | --- | --- | --- |
| *U. japonicus* | G-H36 | HQ564530 | China |
| *U. margarethae* | G-H37 | KY372323 | Beibu Gulf, China |
| *U. margarethae* | G-H38 | KY372322 | Nansha Islands, China |
| *U. moluccensis* | G-H39 | KY372324 | Nansha Islands, China |
| *U. subvittatus* | G-H40 | KY372343 | Nansha Islands, China |
| *U. subvittatus* | G-H40 | KY372342 | Nansha Islands, China |
| *U. subvittatus* | G-H40 | KY372341 | Nansha Islands, China |
| *U. subvittatus* | G-H40 | KY372339 | Nansha Islands, China |
| *U. subvittatus* | G-H40 | KY372337 | Nansha Islands, China |
| *U. subvittatus* | G-H40 | KY372336 | Nansha Islands, China |
| *U. subvittatus* | G-H40 | KY372335 | Nansha Islands, China |
| *U. subvittatus* | G-H40 | KY372333 | Nansha Islands, China |
| *U. subvittatus* | G-H40 | KY372332 | Nansha Islands, China |
| *U. subvittatus* | G-H40 | KY372331 | Nansha Islands, China |
| *U. subvittatus* | G-H40 | KY372329 | Nansha Islands, China |
| *U. subvittatus* | G-H40 | KY372328 | Nansha Islands, China |
| *U. subvittatus* | G-H40 | KY372326 | Nansha Islands, China |
| *U. subvittatus* | G-H41 | KY372340 | Nansha Islands, China |
| *U. subvittatus* | G-H41 | KY372334 | Nansha Islands, China |
| *U. subvittatus* | G-H41 | KY372327 | Nansha Islands, China |
| *U. subvittatus* | G-H42 | KY372338 | Nansha Islands, China |
| *U. subvittatus* | G-H43 | KY372330 | Nansha Islands, China |
| *U. sulphureus* | G-H44 | KY372347 | Northern South China Sea, China |
| *U. sulphureus* | G-H44 | KY372344 | Northern South China Sea, China |
| *U. sulphureus* | G-H44 | EU595334 | South China Sea, China |
| *U. sulphureus* | G-H44 | EU595333 | South China Sea, China |
| *U. sulphureus* | G-H44 | EU595331 | South China Sea, China |
| *U. sulphureus* | G-H44 | EU595330 | South China Sea, China |
| *U. sulphureus* | G-H44 | EU595329 | South China Sea, China |
| *U. sulphureus* | G-H44 | EU595328 | South China Sea, China |
| *U. sulphureus* | G-H44 | EU595327 | South China Sea, China |
| *U. sulphureus* | G-H44 | KY372353 | Beibu Gulf, China |
| *U. sulphureus* | G-H44 | KY372352 | Beibu Gulf, China |
| *U. sulphureus* | G-H44 | KY372351 | Beibu Gulf, China |
| *U. sulphureus* | G-H44 | KY372350 | Beibu Gulf, China |
| *U. sulphureus* | G-H44 | KY372348 | Beibu Gulf, China |
| *U. sulphureus* | G-H44 | KY372345 | Beibu Gulf, China |
| *U. sulphureus* | G-H44 | EF607610 | China |
| *U. sulphureus* | G-H44 | EF607609 | China |
| *U. sulphureus* | G-H45 | EU595332 | South China Sea, China |
| *U. sulphureus* | G-H46 | KY372349 | Beibu Gulf, China |

****Table S3 B** continued**

| **Species** | **Hap ID** | **GenBank accession** | **Sampling locality** |
| --- | --- | --- | --- |
| *U. sulphureus* | G-H47 | KY372346 | Beibu Gulf, China |
| *U. sundaicus* | G-H48 | MZ513962 | Dongfang, Hainan, China |
| *U. tragula* | G-H49 | LC705435 | Guangdong, China |
| *U. tragula* | G-H49 | KY372355 | Lingshui Gulf, China |
| *U. tragula* | G-H49 | KY372354 | Nansha Islands, China |
| *U. tragula* | G-H49 | EF607614 | China |
| *U. tragula* | G-H49 | EF607613 | China |
| *U. tragula* | G-H49 | EF607612 | China |
| *U. tragula* | G-H50 | EF607611 | China |

**Table S4** List of species used in this study with museum numbers and GenBank accession numbers of COI, 16S rRNA, IRBP sequences.

| **Species** | **Sampling locality** | **Museum numbers** | **GenBank accession numbers** | | |
| --- | --- | --- | --- | --- | --- |
|  |  |  | **COI** | **16S rRNA** | **IRBP** |
| *M. flavolineatus* | Xisha Islands, China | MuXS017 | PV834040 | PV833940 | — |
| *M. flavolineatus* | Xisha Islands, China | MuXS018 | PV834041 | PV833941 | PV839439 |
| *M. flavolineatus* | Xisha Islands, China | MuXS019 | PV834042 | PV833942 | PV839440 |
| *M. flavolineatus* | Xisha Islands, China | MuXS024 | PV834043 | PV833943 | PV839441 |
| *M. flavolineatus* | Xisha Islands, China | MuXS025 | PV834044 | PV833944 | — |
| *M. vanicolensis* | Xisha Islands, China | MuXS011 | PV834045 | PV833945 | — |
| *M. vanicolensis* | Xisha Islands, China | MuXS012 | PV834046 | PV833946 | — |
| *M. vanicolensis* | Xisha Islands, China | MuXS013 | PV834048 | PV833947 | PV839442 |
| *M. vanicolensis* | Xisha Islands, China | MuXS014 | PV834047 | PV833948 | PV839443 |
| *M. vanicolensis* | Xisha Islands, China | MuXS026 | PV834049 | PV833949 | PV839444 |
| *P. barberinoides* | Haikou, Hainan, China | GOU102093 | PV834050 | PV833950 | PV839445 |
| *P. barberinus* | Xisha Islands, China | MuSS002 | PV834051 | PV833951 | PV839450 |
| *P. barberinus* | Xisha Islands, China | MuXS001 | PV834052 | PV833952 | PV839446 |
| *P. barberinus* | Xisha Islands, China | MuXS002 | PV834053 | PV833953 | PV839447 |
| *P. barberinus* | Xisha Islands, China | MuXS003 | PV834054 | PV833954 | PV839448 |
| *P. barberinus* | Xisha Islands, China | MuXS004 | PV834055 | PV833955 | PV839449 |
| *P. biaculeatus* | Zhanjiang, Guangdong, China | GOU101521 | PV834056 | PV833959 | PV839453 |
| *P. biaculeatus* | Zhanjiang, Guangdong, China | GOU101523 | PV834058 | PV833960 | — |
| *P. biaculeatus* | Zhanjiang, Guangdong, China | GOU104532 | PV834057 | PV833958 | PV839452 |
| *P. biaculeatus* | Zhanjiang, Guangdong, China | MuZJ003 | PV834059 | PV833956 | PV839451 |
| *P. biaculeatus* | Zhanjiang, Guangdong, China | MuZJ004 | PV834060 | PV833957 | — |
| *P. chrysopleuron* | Zhanjiang, Guangdong, China | GOU101720 | PV834061 | PV833965 | PV839458 |
| *P. chrysopleuron* | Shantou, Guangdong, China | MuST001 | PV834062 | PV833961 | PV839454 |
| *P. chrysopleuron* | Shantou, Guangdong, China | MuST002 | PV834063 | PV833962 | PV839455 |
| *P. chrysopleuron* | Shantou, Guangdong, China | MuST007 | PV834064 | PV833963 | PV839456 |
| *P. chrysopleuron* | Shantou, Guangdong, China | MuST008 | PV834065 | PV833964 | PV839457 |
| *P. ciliatus* | Zhanjiang, Guangdong, China | GOU101644 | PV834066 | PV833966 | PV839459 |
| *P. ciliatus* | Zhanjiang, Guangdong, China | GOU101648 | PV834068 | PV833967 | PV839460 |
| *P. ciliatus* | Xisha Islands, China | GOU104143 | PV834067 | PV833968 | — |
| *P. crassilabris* | Xisha Islands, China | MuXS010 | PV834069 | PV833969 | PV839461 |
| *P. crassilabris* | Xisha Islands, China | MuXS030 | PV834070 | PV833970 | PV839462 |
| *P. cyclostomus* | Xisha Islands, China | MuSS001 | PV834072 | PV833973 | PV839463 |
| *P. cyclostomus* | Xisha Islands, China | MuXS007 | PV834073 | PV833974 | PV839465 |
| *P. cyclostomus* | Xisha Islands, China | MuXS008 | PV834071 | PV833971 | — |
| *P. cyclostomus* | Xisha Islands, China | MuXS009 | PV834074 | PV833972 | PV839464 |
| *P. cyclostomus* | Xisha Islands, China | MuXS031 | PV834075 | PV833975 | — |
| *P. heptacanthus* | Nansha Islands, China | GOU100266 | PV834076 | — | — |
| *P. heptacanthus* | Sanya, Hainan, China | GOU101133 | PV834078 | PV833976 | — |
| *P. heptacanthus* | Shenzhen, Guangdong, China | GOU102172 | PV834079 | PV833977 | PV839467 |

Note: — not available.

**Table S4** **continued.**

| **Species** | **Sampling locality** | **Museum numbers** | **GenBank accession numbers** | | |
| --- | --- | --- | --- | --- | --- |
|  |  |  | **COI** | **16S rRNA** | **IRBP** |
| *P. heptacantha* | Haikou, Hainan, China | MuHK004 | PV834077 | PV833978 | PV839466 |
| *P. heptacantha* | Haikou, Hainan, China | MuHK005 | PV834080 | PV833979 | — |
| *P. indicus* | Xisha Islands, China | XSQD201208 | PV834081 | PV833980 | PV839468 |
| *P. indicus* | Zhuhai, Guangdong, China | GOU102173 | PV834082 | PV833981 | PV839469 |
| *P. indicus* | Zhanjiang, Guangdong, China | GOU102373 | PV834083 | PV833982 | PV839470 |
| *P. indicus* | Zhanjiang, Guangdong, China | GOU102374 | PV834084 | PV833983 | PV839471 |
| *P. indicus* | Xisha Islands, China | GOU104126 | PV834085 | PV833984 | PV839472 |
| *P. multifasciatus* | Xisha Islands, China | XSQD210101 | PV834086 | PV833985 | — |
| *P. multifasciatus* | Xisha Islands, China | XSQD210102 | PV834087 | PV833986 | — |
| *P. multifasciatus* | Xisha Islands, China | XSQD210103 | PV834088 | PV833987 | PV839473 |
| *P. multifasciatus* | Xisha Islands, China | XSQD210104 | PV834089 | PV833988 | PV839474 |
| *P. multifasciatus* | Xisha Islands, China | XSQD210106 | PV834090 | PV833989 | PV839475 |
| *U. heterospinus* | Zhuhai, Guangdong, China | GOU102179 | PV834091 | PV833991 | — |
| *U. heterospinus* | Sanya, Hainan, China | GOU102679 | PV834092 | PV833993 | — |
| *U. heterospinus* | Lingshui, Hainan, China | GOU102044 | PV834093 | — | — |
| *U. heterospinus* | Dongfang, Hainan, China | MuDF001 | PV834094 | PV833990 | PV839476 |
| *U. heterospinus* | Haikou, Hainan, China | MuHK003 | PV834095 | PV833994 | PV839477 |
| *U. heterospinus* | Zhuhai, Guangdong, China | GOU102181 | PV834096 | PV833992 | — |
| *U. heterospinus* | Yangjiang, Guangdong, China | GOU104181 | PV834097 | PV833995 | — |
| *U. heterospinus* | Shantou, Guangdong, China | MuST048 | PV834098 | PV833996 | PV839478 |
| *U. japonicus* | Shanwei, Guangdong, China | GOU102454 | PV834103 | PV834001 | — |
| *U. japonicus* | Sanya, Hainan, China | MuSY004 | PV834105 | PV834004 | PV839483 |
| *U. japonicus* | Sanya, Hainan, China | MuSY007 | PV834104 | PV834005 | PV839484 |
| *U. japonicus* | Shantou, Guangdong, China | MuST029 | PV834106 | PV834002 | PV839485 |
| *U. japonicus* | Shantou, Guangdong, China | MuST031 | PV834107 | PV834003 | PV839486 |
| *U. itoui* | Sanya, Hainan, China | GOU103676 | PV834099 | PV833997 | PV839479 |
| *U. itoui* | Sanya, Hainan, China | GOU103678 | PV834100 | PV833998 | PV839480 |
| *U. itoui* | Yangjiang, Guangdong, China | GOU104100 | PV834101 | PV833999 | PV839481 |
| *U. itoui* | Yangjiang, Guangdong, China | GOU104102 | PV834102 | PV834000 | PV839482 |
| *U. sulphureus* | Yangjiang, Guangdong, China | MuZP009 | PV834108 | PV834006 | PV839487 |
| *U. sulphureus* | Yangjiang, Guangdong, China | MuZP015 | PV834109 | PV834007 | PV839488 |
| *U. sulphureus* | Yangjiang, Guangdong, China | MuZP019 | PV834110 | PV834008 | — |
| *U. sulphureus* | Yangjiang, Guangdong, China | MuZP020 | PV834111 | PV834009 | — |
| *U. sulphureus* | Beibu Gulf, China | MuBBW033 | PV834112 | PV834010 | — |
| *U. sulphureus* | Beibu Gulf, China | MuBBW036 | PV834113 | PV834011 | — |
| *U. sundaicus* | Beihai Guangxi, China | GOU104108 | PV834114 | PV834017 | PV839494 |
| *U. sundaicus* | Zhanjiang, Guangdong, China | GOU104525 | PV834119 | PV834015 | PV839493 |
| *U. sundaicus* | Dongfang, Hainan, China | MuDF004 | PV834115 | PV834012 | PV839489 |
| *U. sundaicus* | Dongfang, Hainan, China | MuDF005 | PV834116 | PV834013 | PV839490 |
| *U. sundaicus* | Haikou, Hainan, China | MuHK001 | PV834117 | PV834016 | PV839491 |

Note: — not available.

**Table S4** **continued.**

| **Species** | **Sampling locality** | **Museum numbers** | **GenBank accession numbers** | | |
| --- | --- | --- | --- | --- | --- |
|  |  |  | **COI** | **16S rRNA** | **IRBP** |
| *U. sundaicus* | Haikou, Hainan, China | MuHK002 | PV834118 | PV834014 | PV839492 |
| *U. tragula* | Haikou, Hainan, China | GOU100443 | PV834120 | PV834018 | — |
| *U. tragula* | Yangjiang, Guangdong, China | GOU100813 | PV834121 | PV834019 | — |
| *U. tragula* | Wenchang, Hainan, China | GOU102011 | PV834122 | PV834020 | — |
| *U. tragula* | Zhuhai, Guangdong, China | GOU102174 | PV834123 | PV834021 | PV839496 |
| *U. tragula* | Zhanjiang, Guangdong, China | GOU102370 | PV834124 | PV834022 | PV839495 |

Note: — not available.

**Table S5** COI haplotype summary for this study (new sequences only).

| ****Hap ID**** | ****Species**** | ****Number**** | **Museum numbers** |
| --- | --- | --- | --- |
| S-H01 | *M. flavolineatus* | 4 | MuXS017 |
| S-H02 | *M. flavolineatus* | 1 | MuXS019 |
| S-H03 | *M. vanicolensis* | 4 | MuXS011 |
| S-H04 | *M. vanicolensis* | 1 | MuXS012 |
| S-H05 | *P. barberinoides* | 1 | GOU102093 |
| S-H06 | *P. barberinus* | 4 | MuSS002 |
| S-H07 | *P. barberinus* | 1 | MuXS001 |
| S-H08 | *P. biaculeatus* | 1 | GOU101521 |
| S-H09 | *P. biaculeatus* | 1 | GOU104532 |
| S-H10 | *P. biaculeatus* | 1 | GOU101523 |
| S-H11 | *P. biaculeatus* | 1 | MuZJ003 |
| S-H12 | *P. biaculeatus* | 1 | MuZJ004 |
| S-H13 | *P. chrysopleuron* | 1 | GOU101720 |
| S-H14 | *P. chrysopleuron* | 1 | MuST001 |
| S-H15 | *P. chrysopleuron* | 2 | MuST002 |
| S-H16 | *P. chrysopleuron* | 1 | MuST008 |
| S-H17 | *P. ciliatus* | 1 | GOU101644 |
| S-H18 | *P. ciliatus* | 1 | GOU104143 |
| S-H19 | *P. ciliatus* | 1 | GOU101648 |
| S-H20 | *P. crassilabris* | 2 | MuXS010 |
| S-H21 | *P. cyclostomus* | 2 | MuXS008 |
| S-H22 | *P. cyclostomus* | 1 | MuXS007 |
| S-H23 | *P. cyclostomus* | 2 | MuXS009 |
| S-H24 | *P. heptacantha* | 5 | GOU100266 |
| S-H25 | *P. indicus* | 1 | XSQD201208 |
| S-H26 | *P. indicus* | 1 | GOU102173 |
| S-H27 | *P. indicus* | 2 | GOU102373 |
| S-H28 | *P. indicus* | 1 | GOU102374 |
| S-H29 | *P. multifasciatus* | 2 | XSQD210101 |
| S-H30 | *P. multifasciatus* | 2 | XSQD210102 |
| S-H31 | *P. multifasciatus* | 1 | XSQD210104 |
| S-H32 | *U. heterospinus* | 4 | GOU102179 |
| S-H33 | *U. heterospinus* | 1 | GOU102679 |
| S-H34 | *U. heterospinus* | 1 | MuHK003 |
| S-H35 | *U. heterospinus* | 1 | GOU104181 |
| S-H36 | *U. heterospinus* | 1 | MuST048 |
| S-H37 | *U. itoui* | 4 | GOU103676 |
| S-H38 | *U. japonicus* | 1 | GOU102454 |
| S-H39 | *U. japonicus* | 2 | MuSY007 |

**Table S5 continued**

| ****Hap ID**** | ****Species**** | ****Number**** | **Museum numbers** |
| --- | --- | --- | --- |
| S-H40 | *U. japonicus* | 1 | MuST029 |
| S-H41 | *U. japonicus* | 1 | MuST031 |
| S-H42 | *U. sulphureus* | 1 | MuZP009 |
| S-H43 | *U. sulphureus* | 4 | MuZP015 |
| S-H44 | *U. sulphureus* | 1 | MuZP020 |
| S-H45 | *U. sundaicus* | 5 | GOU104108 |
| S-H46 | *U. sundaicus* | 1 | MuDF004 |
| S-H47 | *U. tragula* | 1 | GOU100443 |
| S-H48 | *U. tragula* | 1 | GOU100813 |
| S-H49 | *U. tragula* | 1 | GOU102011 |
| S-H50 | *U. tragula* | 1 | GOU102174 |
| S-H51 | *U. tragula* | 1 | GOU102370 |

****Table S6**** Intergeneric and intrageneric genetic distances (%) for Mullidae based on COI(this study+GenBank),16S rRNA(this study), IRBP(this study), and COI+16S rRNA(this study) sequences.

| **Group** | **Geneus** | **Genetic distance (%) COI(this study+GenBank) / 16S rRNA(this study) / IRBP(this study) / COI+16S rRNA(this study)** | | |
| --- | --- | --- | --- | --- |
|  |  | **1** | **2** | **3** |
| 1 | *Mulloidichthys* | 4.43/  1.22/  0.83/  2.63 |  |  |
| 2 | *Parupeneus* | 17.21/  8.27/  11.24/  12.29 | 12.46/  4.76/  1.75/  8.69 |  |
| 3 | *Upeneus* | 18.49/  9.57/  10.27/  14.02 | 19.29/  11.00/  10.37/  14.88 | 11.27/  4.35/  3.19/  7.69 |

****Table S7**** Intraspecific and interspecific genetic distances (%) based on COI (this study+GenBank) sequences.

****Table S7** A** Mulloidichthys

| **Group** | **Species** | **Genetic distance (%)** | |
| --- | --- | --- | --- |
|  |  | **1** | **2** |
| 1 | *M. flavolineatus* | 0.27 |  |
| 2 | *M. vanicolensis* | 7.78 | 0.17 |

****Table S7** B** Parupeneus

| **Group** | **Species** | **Genetic distance (%)** | | | | | | | | | | |
| --- | --- | --- | --- | --- | --- | --- | --- | --- | --- | --- | --- | --- |
|  |  | **1** | **2** | **3** | **4** | **5** | **6** | **7** | **8** | **9** | **10** | **11** |
| 1 | *P. barberinoides* | — |  |  |  |  |  |  |  |  |  |  |
| 2 | *P. barberinus* | 18.69 | 0.17 |  |  |  |  |  |  |  |  |  |
| 3 | *P. biaculeatus* | 18.84 | 13.10 | 0.34 |  |  |  |  |  |  |  |  |
| 4 | *P. chrysopleuron* | 19.42 | 16.45 | 19.18 | 0.32 |  |  |  |  |  |  |  |
| 5 | *P. ciliatus* | 17.39 | 11.90 | 5.14 | 18.02 | 0.17 |  |  |  |  |  |  |
| 6 | *P. crassilabris* | 17.55 | 9.95 | 9.50 | 15.06 | 9.87 | — |  |  |  |  |  |
| 7 | *P. cyclostomus* | 18.51 | 16.25 | 14.08 | 18.08 | 13.54 | 14.15 | 0.17 |  |  |  |  |
| 8 | *P. heptacantha* | 17.07 | 14.00 | 16.68 | 18.10 | 15.94 | 13.43 | 16.32 | 0 |  |  |  |
| 9 | *P. indicus* | 17.11 | 6.47 | 10.62 | 16.91 | 10.01 | 9.39 | 14.56 | 14.74 | 0.43 |  |  |
| 10 | *P. multifasciatus* | 16.45 | 11.29 | 10.28 | 14.19 | 9.81 | 8.98 | 14.04 | 14.11 | 10.87 | 0.24 |  |
| 11 | *P. pleurostigma* | 18.79 | 15.69 | 15.73 | 19.29 | 16.82 | 15.11 | 17.76 | 14.80 | 14.55 | 16.80 | — |

****Table S7 C**** Upeneus

| **Group** | **Species** | **Genetic distance (%)** | | | | | | | | |
| --- | --- | --- | --- | --- | --- | --- | --- | --- | --- | --- |
|  |  | **1** | **2** | **3** | **4** | **5** | **6** | **7** | **8** | **9** |
| 1 | *U. heterospinus* | 0.27 |  |  |  |  |  |  |  |  |
| 2 | *U. itoui* | 10.56 | — |  |  |  |  |  |  |  |
| 3 | *U. japonicus* | 11.55 | 12.45 | 0.64 |  |  |  |  |  |  |
| 4 | *U. margarethae* | 10.20 | 10.41 | 11.41 | 0.17 |  |  |  |  |  |
| 5 | *U. moluccensis* | 17.09 | 15.88 | 17.67 | 17.18 | — |  |  |  |  |
| 6 | *U. sulphureus* | 18.23 | 17.36 | 18.07 | 18.24 | 12.70 | 1.04 |  |  |  |
| 7 | *U. sundaicus* | 10.23 | 9.27 | 12.08 | 10.97 | 15.27 | 18.85 | 0.11 |  |  |
| 8 | *U. tragula* | 9.62 | 9.13 | 10.72 | 7.96 | 15.67 | 16.66 | 8.98 | 0.23 |  |
| 9 | *U. subvittatus* | 18.26 | 16.88 | 18.30 | 17.52 | 7.93 | 12.79 | 16.26 | 16.55 | 0.25 |

Note: “—” indicates that intraspecific genetic distance could not be calculated because only one sequence (n = 1) was available for the species

****Table S8**** Intraspecific and interspecific genetic distances (%) based on 16S rRNA, IRBP, and COI+16S rRNA sequences in this study.

****Table S8** A** Mulloidichthys

| **Group** | **Species** | **Genetic distance (%) 16S rRNA/ IRBP/ COI+16S rRNA** | |
| --- | --- | --- | --- |
|  |  | **1** | **2** |
| 1 | *M. flavolineatus* | 0/  0.08/  0.07 |  |
| 2 | *M. vanicolensis* | 2.17/  1.34/  4.67 | 0.08/  0.08/  0.07 |

****Table S8** B** Parupeneus

| **Group** | **Species** | **Genetic distance (%) 16S rRNA/ IRBP/ COI+16S rRNA** | | | | | | | | | |
| --- | --- | --- | --- | --- | --- | --- | --- | --- | --- | --- | --- |
|  |  | **1** | **2** | **3** | **4** | **5** | **6** | **7** | **8** | **9** | **10** |
| 1 | *P. barberinoides* | — |  |  |  |  |  |  |  |  |  |
| 2 | *P. barberinus* | 4.33/  2.65/  11.88 | 0/  0/  0.03 |  |  |  |  |  |  |  |  |
| 3 | *P. biaculeatus* | 6.43/  1.90/  12.46 | 2.78/  2.03/  8.35 | 0.08/  0.24/  0.20 |  |  |  |  |  |  |  |
| 4 | *P. chrysopleuron* | 5.15/  2.70/  13.42 | 5.62/  2.06/  11.16 | 6.55/  1.82/  12.72 | 0.15/  0.10/  0.18 |  |  |  |  |  |  |
| 5 | *P. ciliatus* | 5.35/  2.01/  11.86 | 1.94/  2.14/  7.39 | 0.81/  0.78/  3.04 | 6.52/  1.68/  12.18 | 0/  0/  0.19 |  |  |  |  |  |
| 6 | *P. crassilabris* | 9.19/  2.01/  12.11 | 2.33/  2.14/  6.19 | 3.16/  1.00/  6.77 | 6.27/  1.93/  10.54 | 2.73/  1.25/  6.69 | 0/  0/  0.08 |  |  |  |  |
| 7 | *P. cyclostomus* | 5.77/  2.44/  14.43 | 7.93/  2.05/  11.89 | 10.21/  1.42/  12.25 | 11.78/  1.72/  14.80 | 9.27/  1.67/  11.55 | 9.03/  1.42/  11.50 | 0/  0.08/  0.08 |  |  |  |
| 8 | *P. heptacantha* | 4.62/  2.91/  11.55 | 2.72/  2.52/  8.16 | 4.59/  2.42/  10.36 | 7.10/  2.58/  12.59 | 3.73/  2.53/  9.70 | 3.93/  2.53/  8.55 | 8.79/  2.44/  12.25 | 0/  0/  0 |  |  |
| 9 | *P. indicus* | 5.35/  2.91/  11.24 | 1.04/  0.50/  3.97 | 3.05/  2.16/  7.08 | 5.76/  2.19/  11.38 | 2.22/  2.26/  6.49 | 2.21/  2.27/  5.90 | 8.25/  2.18/  11.00 | 2.61/  2.65/  8.23 | 0.11/  0/  0.25 |  |
| 10 | *P. multifasciatus* | 12.02/  2.52/  11.20 | 3.12/  2.39/  7.18 | 2.96/  1.50/  7.08 | 5.42/  2.19/  9.80 | 2.92/  1.75/  6.85 | 2.52/  1.00/  5.69 | 9.89/  1.67/  11.63 | 4.12/  2.78/  8.77 | 3.00/  2.52/  6.81 | 0/  0/  0.08 |

****Table S8 C**** Upeneus

| **Group** | **Species** | **Genetic distance (%) 16S rRNA/ IRBP/ COI+16S rRNA** | | | | | |
| --- | --- | --- | --- | --- | --- | --- | --- |
|  |  | **1** | **2** | **3** | **4** | **5** | **6** |
| 1 | *U. japonicus* | 0.08/  0/  0.31 |  |  |  |  |  |
| 2 | *U. heterospinus* | 2.38/  1.71/  6.83 | 0/  0.17/  0.09 |  |  |  |  |
| 3 | *U. itoui* | 2.33/  2.14/  7.05 | 1.40/  2.10/  5.92 | 0.10/  0/  0.04 |  |  |  |
| 4 | *U. sulphureus* | 8.67/  7.88/  13.21 | 9.60/  7.86/  13.62 | 9.55/  7.48/  13.33 | 0/  0/  0.27 |  |  |
| 5 | *U. sundaicus* | 3.17/  3.69/  7.43 | 2.72/  3.66/  6.34 | 2.28/  3.57/  5.80 | 9.60/  6.36/  14.14 | 0/  0/  0.03 |  |
| 6 | *U. tragula* | 2.98/  2.14/  6.45 | 2.53/  2.10/  6.10 | 1.99/  1.50/  5.46 | 10.49/  7.34/  13.57 | 3.53/  3.31/  6.25 | 0/  0/  0.20 |

Note: “—” indicates that intraspecific genetic distance could not be calculated because only one sequence (n = 1) was available for the species

****Table S9**** Sequence identity check and unified haplotype collapsing for six Upeneus sulphureus COI haplotypes grouped in the phylogeny.

| **Original Hap ID** | **Accession** | **Sampling locality** | ****COI clade in Figure 3**** | ****Unified Hap ID**** |
| --- | --- | --- | --- | --- |
| G-H46 | KY372349 | Beibu Gulf, China | Clade 1 | Hap 1 |
| G-H47 | KY372346 | Beibu Gulf, China | Clade 1 | Hap 2 |
| G-H45 | EU595332 | South China Sea, China | Clade 2 | Hap 3 |
| G-H44 | KY372347 | Northern South China Sea, China | Clade 2 | Hap 4 |
| S-H43 | MuZP015 | Yangjiang, Guangdong, China | Clade 2 | Hap 4 |
| S-H42 | MuZP009 | Yangjiang, Guangdong, China | Clade 2 | Hap 5 |

**Note:** This table provides a **sequence identity check** and **unified haplotype collapsing** for six Upeneus sulphureus COI haplotypes that formed two tight subclades in the phylogeny. COI sequences from this study and GenBank were merged, aligned, and trimmed to a common overlapping region; records with **identical sequences (100% identity)** in the merged dataset were collapsed into the same **Unified Hap ID**. “Original Hap ID” denotes haplotype codes from the prior source-specific haplotype screenings, where the **G-** prefix indicates GenBank-derived haplotype IDs and the **S-** prefix indicates haplotype IDs from this study. “Accession” refers to the GenBank accession number or the internal sample/sequence ID for newly generated sequences. “Sampling locality” is reported as provided in GenBank metadata or recorded for specimens in this study. COI clade assignment follows the COI phylogeny in Figure 3.
